# The Cytochrome *bd* Oxidase of *Porphyromonas gingivalis* Contributes to Oxidative Stress Resistance and Dioxygen Tolerance

**DOI:** 10.1371/journal.pone.0143808

**Published:** 2015-12-02

**Authors:** Julia Leclerc, Eric Rosenfeld, Mathieu Trainini, Bénédicte Martin, Vincent Meuric, Martine Bonnaure-Mallet, Christine Baysse

**Affiliations:** 1 EA1254 Microbiologie—Risques Infectieux, University of Rennes1, Rennes, France; 2 UMR CNRS 7266 LIENSs, University of La Rochelle, La Rochelle, France; 3 CHU Rennes, Rennes, France; East Carolina University School of Medicine, UNITED STATES

## Abstract

*Porphyromonas gingivalis* is an etiologic agent of periodontal disease in humans. The disease is associated with the formation of a mixed oral biofilm which is exposed to oxygen and environmental stress, such as oxidative stress. To investigate possible roles for cytochrome *bd* oxidase in the growth and persistence of this anaerobic bacterium inside the oral biofilm, mutant strains deficient in cytochrome *bd* oxidase activity were characterized. This study demonstrated that the cytochrome *bd* oxidase of *Porphyromonas gingivalis*, encoded by *cydAB*, was able to catalyse O_2_ consumption and was involved in peroxide and superoxide resistance, and dioxygen tolerance.

## Introduction

*Porphyromonas gingivalis* is a gram negative anaerobe populating the oral cavity. *P*. *gingivalis* resides in the dental plaque and it is a main contributor to periodontal diseases. The oral biofilm is a model of microbial multicellularity and multicellular behaviour ranging from commensal microbiome to virulent infection. The accumulated body of studies of bacterial pathogens in periodontal acute and chronic infections have designated *P*. *gingivalis*, *Tannerella forsythia* and *Treponema denticola* (also called “the red complex”) as the tripartite cornerstone of the community in its pathogenic state, as recently confirmed by metagenomic microbiome analysis [1]. Not only do they produce proteases, toxins and inflammatory compounds that attack host tissue, but they can shape the whole behaviour of the community [2]. Nevertheless, the physiological properties of each individual of the red complex are not fully understood. In this respect, *P*. *gingivalis* remains a puzzling organism. Although it is non-motile, and possesses a rather undersized armament for adaptive responses compared to most ubiquitous organisms, *P*. *gingivalis* sustains the infection disease by surviving in the unfavourable environment of the oral cavity and periodontal pockets, and by invading host tissues. For example, the *P*. *gingivalis* ATCC 33277 genome encodes only four two-component systems, one orphan histidine kinase, two orphan response regulators, one histidine kinase/response regulator hybrid protein [3] and six extracytoplasmic sigma factors, including SigH (PGN_1740) which is required for resistance to oxygen and oxidative stress [4–6]. As reviewed by Henry *et al*. [7], the apparent robustness of *P*. *gingivalis* is largely promoted by its ability to circumvent oxidative stress via intracellular enzymes that detoxify the ROS originating from many host-dependent and environment-dependent processes in the oral cavity. Surprisingly, this anaerobic organism possesses an O_2_-dependent respiratory enzyme. CydAB is a prokaryote-specific membrane-bound oxidase, with two heme *b* cofactors and one heme *d*, which is considered as the site for oxygen reduction [8]. Thanks to its high affinity for oxygen, CydAB is used for energy generation in oxygen-limiting conditions by facultative anaerobes, and by nanaerobes. This term, ‘nanaerobe’, was implemented to designate bacteria whose cell population grows optimally in an anaerobic environment but which are able to multiply in the presence of oxygen, such as *Bacteroides fragilis* [9]. In addition to promoting O_2_-dependent growth, CydAB is involved in the detoxification of ROS and RNS, and promotes survival during the stationary phase of growth, in some bacteria such as *Escherichia coli*, *Azotobacter vinelandii* and *Shewanella oneidensis* [10–12]. In *E*. *coli*, the cytochrome *bd* displayed catalase activity: the purified enzyme catalyzed efficiently the decomposition of H_2_O_2_ with formation of O_2_. Moreover, cyanide, which binds ferric heme *d*, was found to inhibit the catalase activity. The authors also demonstrated that overexpressing *cydAB* in *E*. *coli* resulted in transformation of H_2_O_2_ in O_2_ in catalase-minus *E*. *coli* mutants [13]. The involvement of CydAB in maintaining the redox metabolism of bacterial cells was also demonstrated in *E*. *coli*, for which CydAB was described as an electron sink during periplasmic disulphite oxidation, or as a supply of oxidative power for haem synthesis [14, 15]. CydAB also decreases electrons availability for the fumarate reductase Frd. In *E*. *coli*, the fumarate reductase Frd is an important source of H_2_O_2_ in aerobic conditions; CydAB diminishes the rate of H_2_O_2_ formation by Frd when anaerobic cultures are exposed to O_2_ [16].

In *P*. *gingivalis* the main respiratory enzyme is Frd, which supports anaerobic energy generation. Chemical inhibition of Frd has been shown to diminish growth rate and biofilm formation [17]. No *P*. *gingivalis* mutant with altered *frdAB* genes has been reported nor studied, but in *Bacteroides fragilis* such a mutant grew very poorly [18]. The role of CydAB in *P*. *gingivalis* remains unexplored, except as regards the unaltered biofilm forming ability of the *cydAB* mutant [19]. Transcriptomic data have only revealed that *cydAB* gene expression was upregulated by oxygen [20] and negatively controlled by a LuxS homolog [21]. Focusing on *P*. *gingivalis*, this work examines the implications of CydAB for stress response and adaptation, and for interaction with host cells. These data lead us to rethink the classification of *P*. *gingivalis*, which has long been considered as a strict anaerobe in regard to its O_2_ metabolism. Indeed a new classification was proposed by Morris and Schmidt for bacteria that can harvest O_2_ present in nanomolar concentrations [22].

## Methods

### Bacterial strains and growth conditions

*Porphyromonas gingivalis* ATCC 33277 was cultured in enriched BHI broth containing, per liter, 37 g of BHI powder (AES Chemunex, France), 5 g of yeast extract (Conda, Dutscher) 25 mg of hemin (Sigma), and 10 mg of menadione (Sigma)_._ The strains were maintained on Columbia 3 agar plates supplemented with 5% (v/v) defibrinated horse blood (AES Chemunex, Combourg, France), 25 mg/l of hemin, and 10 mg/l of menadione. The cultures were incubated at 37°C in an anaerobic chamber Macs-VA500 (Don Whitley) flooded with 80% N_2_, 10% H_2_ and 10% CO_2._ However, brief exposures to oxygen outside the chamber cannot be avoided for some experiments. The media were supplemented with erythromycin 5 μg/ml, and tetracycline 1 μg/ml when required. The *P*. *gingivalis cydAB* deletion mutant was constructed and is described by Leclerc *et al*. [19]. This mutant is resistant to erythromycin. The exact role of the *cydW* gene that is part of the *cydWAB* operon is unknown. Therefore we have not deleted the gene to study the phenotype of a cytochrome *bd* oxidase mutant.

### Complementation of the *cydAB* mutant with the native genes *in trans*

*In trans* complementation often leads to an overexpression of the genes. Overexpressing *cydAB* without overexpressing *cydW* may confer pleiotropic phenotypes, if CydW is involved for example in the correct folding or stabilisation of the cytochrome *bd* oxidase inside the inner membrane. Therefore we used a 3 425 bp DNA fragment containing the 3 ORF of the *cydWAB* operon (PGN_1040 to PGN_1042) and the 179 bp 5’ intergenic region between PGN_1039 and PGN_1040, that has been amplified by PCR using the primers CydAB1 and CydAB2 (Table 1). The fragment digested by *Sph*I (NEB England) was then ligated into the *Sph*I linearised vector pYKP028 [23], and the insert sequence was verified by nucleotide sequencing and *in silico* comparison with the published genome sequence [3]. The resulting vector pYKP028_cydWAB, conferring tetracycline resistance, was introduced into a *P*. *gingivalis cydAB* mutant by electroporation as previously described [24]. Complemented mutants were selected by their combined resistance to erythromycin and tetracycline. The expression of *cydWAB* genes from the recombinant plasmid was verified by RT-PCR. The nucleotide sequence of the DNA insert was verified compared to the native genomic operon.

**Table 1:** Primers for RT-PCR and qRT-PCR.

Name	Sequence
CydAB1	GCATGCGAGGGACATTGCTGGTATTG
CydAB2	GCATGCATTGCTATCGTTTGGTCAGG
PGN_0564-L (*sodB*)	AATTCCACCACGGTAAGCAC
PGN_0564-R (*sodB*)	GAGCCGAATTGTTTGTCGAT
PGN_0381-L *(glk)*	ATGAATCCGATCCGCCACCAC
PGN_0381-R *(glk)*	GCCTCCCATCCCAAAGCACT
PGN_1040-L (*cydW*)	GGGAAAGACGCTATGGGCTA
PGN_1041-L (*cydA*)	ACACTGGGATTGGGTGTCAT
PGN_1041-R (*cydA*)	CACCGATCGCAAAGTTGA
PGN_1042-R (*cydB*)	TCCTCCCCCATACATTACCA

### ROS (Reactive Oxygen Species) resistance assay

For H_2_O_2_ survival assays, *P*. *gingivalis* strains (wild-type, *cydAB* mutant and *cydAB* complemented mutant) were grown overnight in enriched BHI at OD_600 nm_ of 0.5 (exponential phase), and 1.8 (stationary phase). For paraquat survival assays, the three *P*. *gingivalis* strains were grown overnight in enriched BHI at OD_600 nm_ of 0.5 (exponential phase). Samples of these cultures were used to inoculate an enriched BHI medium to an OD_600 nm_ of 0.1. Each culture was split in two, and one half was treated with 500 μM of H_2_O_2_ (Sigma) or 480 μM of paraquat (Sigma). The other half of each culture was left untreated to serve as a control. 10 μl of each sample was removed at 0, 1, 2 and 3 h 30 (for H_2_O_2_ treatment) or at 0, 1, 2, 4 and 6 h (for paraquat treatment) of incubation at 37°C in anaerobic conditions (incubation in the anaerobic chamber, but addition of paraquat and sampling for CFU-counting outside the chamber i.e. exposed to atmospheric oxygen) or in aerobic conditions. Serial dilutions were made in duplicate with enriched BHI broth. An aliquot of each dilution was spread on Colombia agar plates supplemented with blood. Colonies were enumerated after five days of anaerobic incubation at 37°C. At least three independent experiments, each in duplicate or triplicate, were conducted.

### Dioxygen tolerance

Bacterial cells from overnight cultures in enriched BHI of *P*. *gingivalis* (wild-type, *cydAB* mutant and *cydAB* complemented mutant) at OD_600nm_ 0.5 were washed and suspended in phosphate buffered saline (PBS) supplemented with 5 g/l of yeast extract to an OD_600 nm_ of 0.1.The CFU/ml of each culture was determined after incubation for six hours at 37°C in aerobic conditions, with shaking at 150 rpm, or in anaerobic conditions. Serial dilutions were made and were spread on supplemented Colombia agar plates. Colonies were enumerated after five days of anaerobic incubation at 37°C.

### RT-PCR and qRT-PCR

Overnight cultures of *P*. *gingivalis* (wild-type and *cydAB* mutant) at OD_600 nm_ 0.5 (exponential phase) grown in enriched BHI were used to inoculate enriched BHI cultures to OD_600 nm_ 0.1. Each culture was split in two, and one half was treated with 320 μM of Paraquat (Sigma). After one hour at 37°C in an anaerobic atmosphere, samples were harvested by centrifugation (4000 rpm for 15 min at room temperature). Bacterial lysis and RNA extraction were performed with mirVana^TM^ miRNA isolation kit (Ambion, Life technologies) according to the manufacturer’s instructions. Residual DNA was removed by DNase treatment using the Turbo DNA-free (Ambion, Life technologies) and extraction was purified with the RNA Clean & Concentrator^TM^-5 (Zymo Research, Proteigen) according to the manufacturer’s instructions. The quantity of the extracted RNA was confirmed with a NanoDrop spectrophotometer (Thermo Scientific). The amount of RNA was estimated by determining the absorbance at 260 nm.

Real-Time quantitative Polymerase Chain Reaction (qRT-PCR) was used to assess the level of expression of selected *P*. *gingivalis* genes. A reverse transcription reaction was performed with the M-MLV reverse transcriptase (Promega) using random hexamer primers, according to the manufacturer’s recommendations.

Conventional PCR was performed using the OneTaq HS enzyme (New Englands BioLabs) according to the manufacturer’s protocol, using a BioRad C1000 thermal cycler.

qRT-PCR assays were performed using SYBR green/ROX chemistry (Eurogentec) with the ABI Prism 7000 Sequence Detection System and software (Applied Biosystem). The differences in messenger RNA expression, Ct (Cycle threshold) value, were calculated by the 2^-ΔΔCt^ method in which the amount of target RNA was adjusted to the amount of a reference internal RNA, the *glk* transcript (PGN_0381). Real-time quantitative PCR was carried out for each gene in triplicate from two independent experiments. The primers used in this experiment are described in Table 1.

### Analysis of O_2_ consumption by high resolution respirometry

Oxygen concentration was measured by high-resolution respirometry with an Oroboros Oxygraph-2k (Oroboros Instruments), in a standard configuration, with 2 ml volume of both chambers, at 37°C, and 500 rpm stirrer speed. *P*. *gingivalis* cells grown at 37°C in anaerobic conditions (Anaerogen, Oxoid) were harvested in the exponential growth phase (OD_600 nm_ 0.5), washed or not in PBS according to the experimental conditions tested, and transferred into oxygraph chambers. Acquisition was started after 2–3 minutes of equilibration at 37°C in oxic conditions and after closing of the chambers. Measurements were performed on two independent cultures, and the data shown represent typical results. Data were recorded at 1 s intervals using the Datlab 4 Acquisition software (Oroboros, Innsbruck, Austria). Standardized calibration procedures of the oxygen signal were carried out using the enriched BHI medium or PBS. Respiration was automatically corrected for contributions of the polarographic oxygen sensor and of oxygen diffusion to total apparent respiration, as a continuous function of oxygen concentration.

### Adhesion and Invasion into epithelial cells Ca9-22

Epithelial cells Ca9-22 (Japanese Collection of Research Bioresources Cell Bank, JCRB) were cultivated at 37°C in aerobiosis with 5% CO_2_ in humidified atmosphere in Eagles’ Modified Essential Medium (EMEM) enriched with 10% (v/v) fetal calf serum, 1% (v/v) of 200 mM L-glutamine and supplemented or not with 1% (v/v) of an antibiotic mixture containing 10 U/μl penicillin and 10 U/μl streptomycin. Epithelial cells Ca9-22 were seeded into 24-well culture plates at a density of approximately 1,5 10^5^ cells per well. Cells were grown to confluent monolayers over 48 hours and washed twice with 500 μl of PBS. *P*. *gingivalis* strains (wild-type, *cydAB* mutant and complemented *cydAB* mutant) were incubated with Ca 9–22 cells with a multiplicity of infection of about 1:1000, for 30 minutes at 37°C in aerobiosis with 5% CO_2_ in humidified atmosphere in enriched -EMEM without antibiotic. After incubation, unattached bacteria were removed by three washes with PBS.

To evaluate the percentage (compared to inoculum) of bacteria that are associated to Ca9-22 (both adherent and internalized bacterial cells), samples were plated on Colombia agar medium supplemented with blood.

To evaluate the percentage of bacteria which have been internalized into Ca9-22 cells, samples were incubated for 1 hour in enriched EMEM (i.e. with antibiotics to kill external bacteria) at 37°C with 5% CO_2_ in humidified atmosphere. External adherent bacteria were removed by three PBS washes and the samples were plated on Colombia agar medium supplemented with blood.

Colonies were enumerated after 5 days of incubation at 37°C, in anaerobic conditions. At least three independent experiments, each in duplicate, were conducted.

## Results and Discussion

### The *cydWAB* genes are transcribed as one polycistronic mRNA

In *E*. *coli*, the cytochrome *bd* oxidase complex is encoded by three genes, from which the last one, *cydX*, was recently found to be essential for the heme-bound active site stability [25, 26]. Over 300 homologues of *cydX* were identified in bacteria, including 80 unannotated genes [27]. The sequence analysis of these proteins shows a great degree of variability, with only a few highly-conserved residues. Sequenced genomes of *P*. *gingivalis* (http://www.ncbi.nlm.nih.gov/genome/) do not encode the *cydX* homologue. By further investigation of *cydAB* operons in bacterial genomes, Allen *et al*., (2014) identified two additional conserved hypothetical small proteins encoded at the 3’end of some operons: CydY and CydZ [27]. We have not found any *cydY* or *cydZ* homologues immediately downstream of *cydAB* in *P*. *gingivalis* genomes.

**Fig 1:**
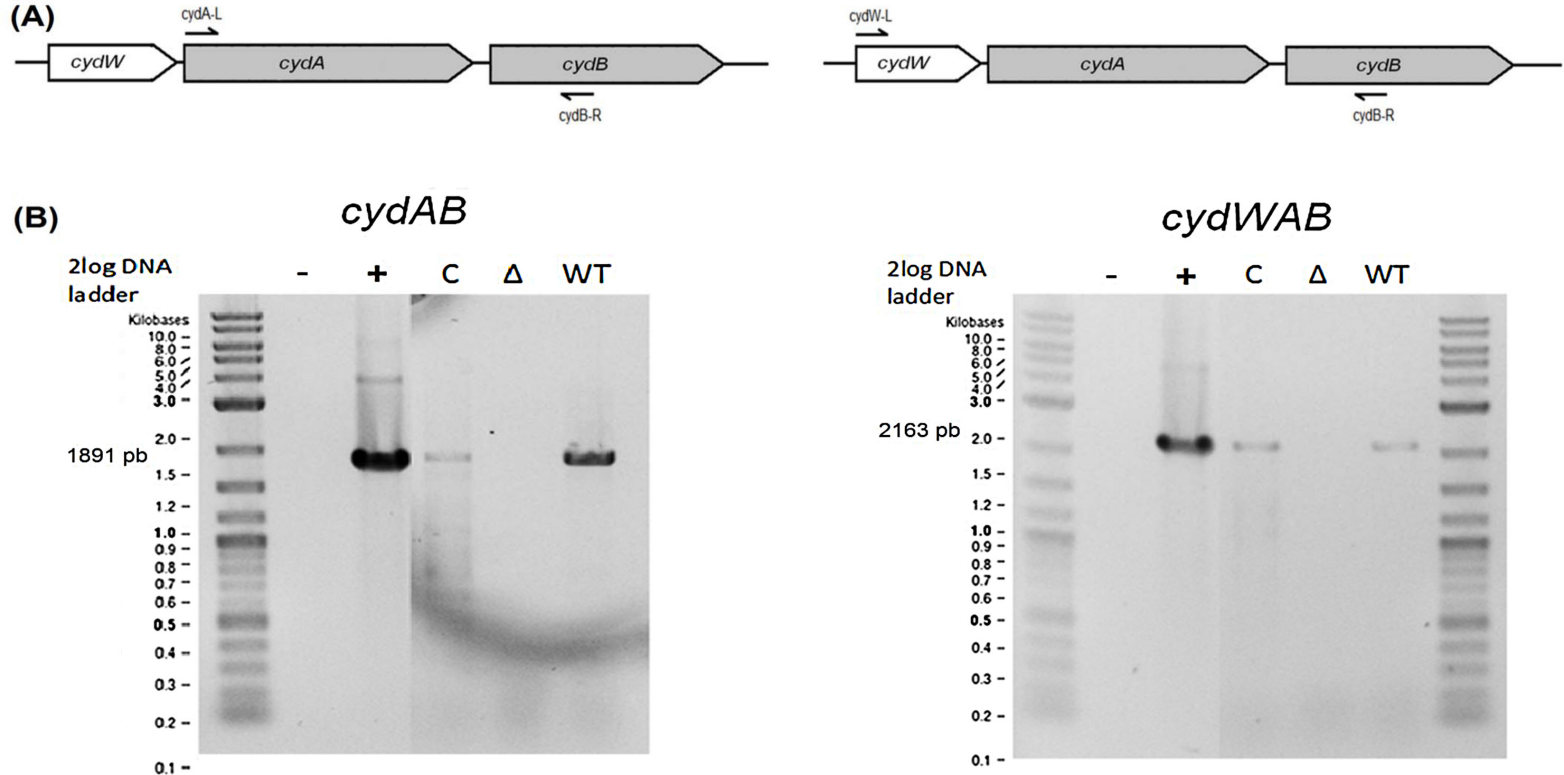
Organisation of the *cydWAB* genes. **(A)** Map of *cydWAB* operon and the positions of primers used for PCR. To construct the *cydAB* mutant used in this study, a DNA fragment containing part of both *cydA* and *cydB* (in grey) was deleted. **(B)** PCR performed on cDNA obtained by reverse transcription on total RNA extract from *P*. *gingivalis* ATCC 33277 (WT), *cydAB* mutant (Δ) and complemented mutant (C). H_2_O and genomic DNA of *P*. *gingivalis* ATCC 33277 were used as controls.

In the *P*. *gingivalis* ATCC 33277 genome, the *cydAB* genes are located downstream of an unannotated ORF, that we called *cydW* (PGN_1040) (**Fig 1A**). It encodes a hypothetical membrane protein of 82 amino acids, and a search was performed using the Blastp program to compare the proteins of the NR database to the CydW sequence. CydW-homologues are widely distributed amongst the members of bacteroidales family (**S1 Fig**). The function of this putative membrane protein is unknown.

By RT-PCR analyses, we demonstrated that *cydWAB* genes are part of the same operon (**Fig 1B**) in the wild type strain. Additional controls are displayed in the supporting information (**S2 Fig)**. In the *cydAB* mutant we verified that the transcription of the *cydAB*-downstream gene (PGN_1043) and *cydW* was not defective due to the mutation (data not shown). Therefore, we proved that the c*ydAB* mutant contained a non-polar deletion of the central part of the *cydWAB* operon (**Fig 1B**). Moreover, we found that the *cydWAB* operon was transcribed at both exponential and stationary phases of growth in the wild-type strain, in anaerobic condition, although LuxS was reported as a repressor of *cydAB* in anaerobiosis [21].

**Fig 2:**
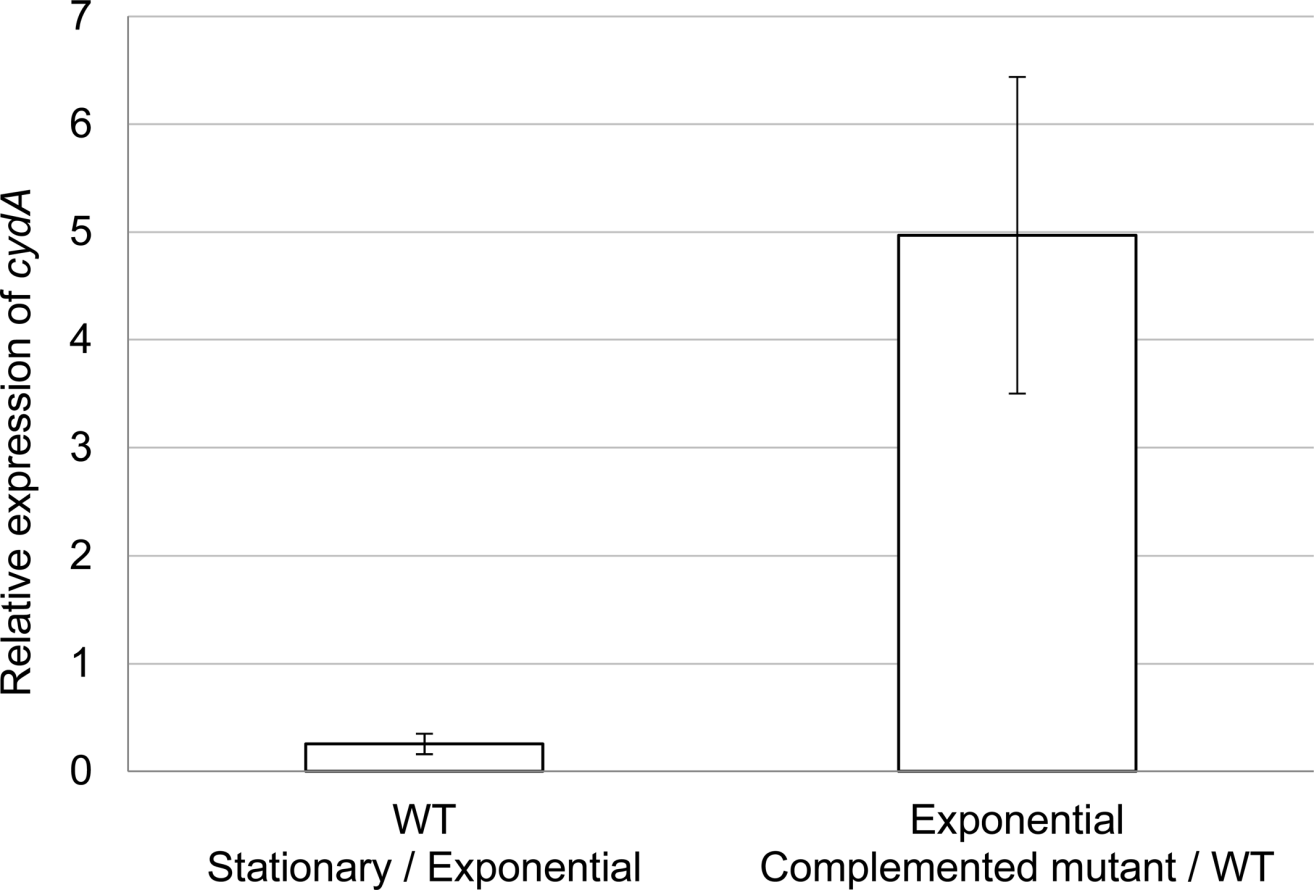
Relative expression of *cydA*. The relative expression was quantified by qRT-PCR with the 2^­∆∆Ct^ method. The normalization was done with the glucokinase house-keeping gene (PGN_0381). The expression of *cydA* in the wild-type strain was compared between stationary and exponential phases of growth. The white bar represents the ratio of expression in the stationary phase vs. the exponential phase.The relative expression of *cydA* in the exponential phase was compared between the complemented *cydAB* mutant and the wild type strains. The grey bar represents the ratio of expression of complemented mutant vs. wild-type. These data are the mean and standard deviations of two biological replicates containing three technical replicates.

By quantitative RT-PCR, we saw that the expression of *cydA* was four times higher in the exponential phase of growth than in the stationary phase of growth (**Fig 2**) in *P*. *gingivalis* ATCC 33277. Therefore, survival assays and respirometry assays were carried out with exponentially growing cultures.

### A *trans* complementation restores the *cydWAB* transcription

We verified that the *cydWAB* operon was transcribed in the *cydAB* mutant carrying the *cydWAB* genes encoded by the pYKP028_cydWAB vector. However, the complemented mutant not only expressed but overexpressed the *cydA* gene: during the exponential growth phase, expression was over five times the expression of *cydA* in a wild type strain (**Fig 2**), and over twelve times at the stationary phase of growth (data not shown), as monitored by qRT-PCR analyses. In the complemented mutant the expression of *cydA* was constant throughout the growth phases (data not shown). This result may explain the inability of the complemented mutant to behave exactly as the wild type in stress survival assays and respirometry experiments, as described in the following chapters.

### The *cydAB* mutant is more sensitive to oxidative stress

#### Superoxide donor

Paraquat was used to generate superoxide anion radicals. Paraquat undergoes redox cycling *in vivo*, being reduced by electron donors such as NADPH, and it needs to be oxidized by an electron receptor such as dioxygen to produce superoxide. In *E*. *coli*, paraquat can also alter the intracellular NADPH/NADP+ ratio by diverting electron flow [28] and can induce the SoxRS stress response [29]. Therefore, susceptibility to paraquat depends either on detoxification efficiency or on the intracellular concentration of the electron receptor that is required to generate superoxide. O_2_°^-^ is unable to cross the cell envelope; therefore, as previously demonstrated in *E*. *coli*, the only O_2_°^-^ which is effective is that generated within the cell by paraquat [30]. Detection of intracellular ROS, using redox probes or agents, may be a source of artefacts. Such detection was discarded in the present work because of the possibilities of interference with paraquat and menadione (from the enriched BHI medium that is used for growth) and the difficulty controlling O_2_ levels at very low concentrations.

**Fig 3:**
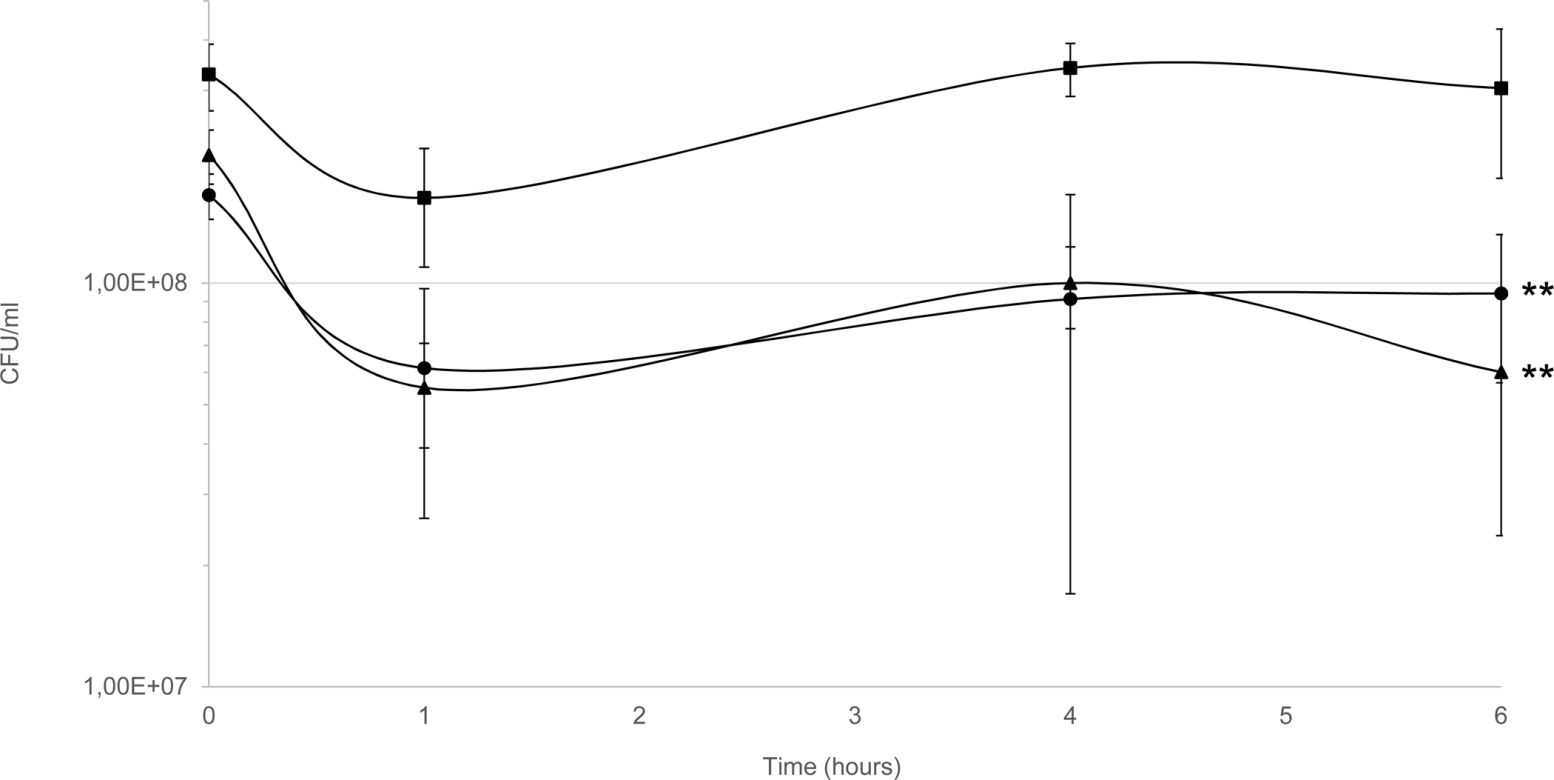
Survival assay with paraquat, a superoxide generator. The effect of paraquat on the viability of *P*. *gingivalis* wild-type, *cydAB* mutant and complemented mutant was tested by exposing the strains to 480 μM of paraquat in enriched BHI medium at 37°C in anaerobic condition, and determining the CFU/ml at different time points for six hours. Wild-type (■), *cydAB* mutant (▲) and complemented *cydAB* mutant (●). Bars represent standard errors and asterisks indicate statistically significant differences between values of wild-type strain and mutant or complemented mutant (** p<0.01). Statistical analysis were performed with GraphPad Prism software (California, USA). The Mann-Whitney test was used and p-values<0.05 were considered significant.

The *P*. *gingivalis* wild-type, *cydAB* mutant and complemented mutant were exposed to an inhibitory (i.e. causing no visible growth after 24 h of incubation) concentration of paraquat (480 μM) in anaerobic conditions following a short exposure to oxygen during paraquat addition and the surviving cells were quantified. The percentages of surviving cells of wild-type, mutant and complemented mutant were respectively 49, 26 and 39%, relative to the untreated samples, after one hour of treatment (**Fig 3**). The differences in susceptibility between wild-type and mutant suggest that *cydAB* is either involved in superoxide detoxification or in removing the residual electron acceptor O_2_ required for the production of ROS from paraquat. When the experiment was performed in aerobic conditions with the same concentration of paraquat, the survival of the *cydAB* mutant was not affected compared to WT (data not shown). In anaerobiosis, restoring *cydAB* expression in *trans* partially restores the wild-type phenotype in the *cydAB* mutant (**Fig 3**). However, the reason why overexpressing the *cyd* operon by plasmid-based complementation did not totally restore the resistance of the mutant to paraquat is not understood.

**Fig 4:**
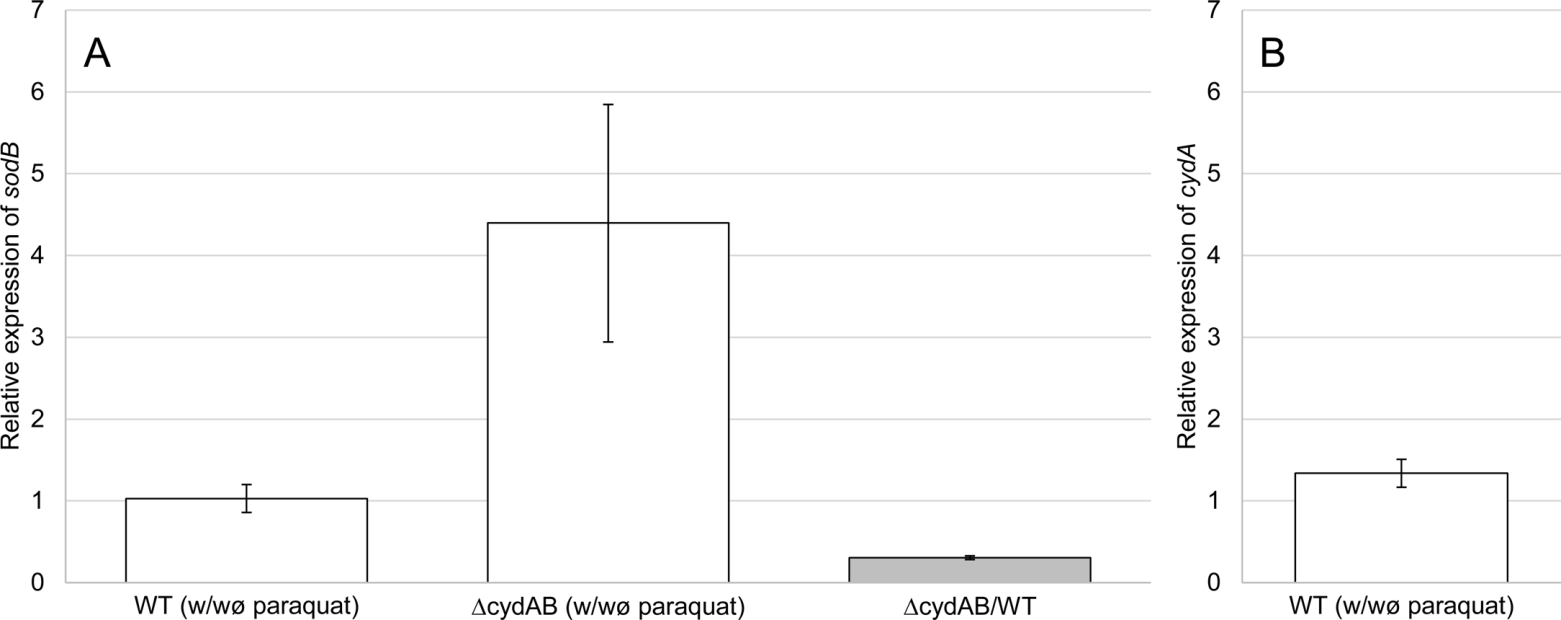
Relative expression of *sodB*. The relative expression of *sodB* was quantified by qRT-PCR with the 2^­∆∆Ct^ method. The normalization was done with the glucokinase gene (PGN_0381). The white histograms represent, for wild-type or *cydAB* mutant strains, the relative expression of *sodB* **(A)** or *cydA* **(B)** between both conditions: with paraquat (320 μM) and without paraquat (control condition). The grey histogram represents the relative expression of *sodB* between *cydAB* mutant and wild-type (control condition) in absence of paraquat. Bars represent standard errors.

The expression of *sodB* (PGN_0564) encoding the superoxide dismutase was monitored in *P*. *gingivalis* wild type and *cydAB* mutant. In *E*. *coli* redox-cycling drugs were able to activate the SoxR (*sodB* activator) in anaerobic cells, thus without superoxide production, as long as alternative respiratory acceptors were provided [31]. In *P*. *gingivalis*, there is no SoxRS system. *sodB* expression is activated by exposure to oxygen [20] and by paraquat in aerobiosis [32]. qRT-PCR analyses showed that the expression of *sodB* was not induced by a sub-inhibitory concentration of paraquat (320 μM) in the wild-type strain, while the expression was four times higher in the *cydAB* mutant (**Fig 4A**). Without paraquat, and in anaerobic conditions, the expression level of *sodB* was three times lower in the *cydAB* mutant than in the wild-type (**Fig 4A**). One hypothesis is that CydAB decreased paraquat toxicity in anaerobiosis by consuming the trace of O_2_ resulting from the short exposure to air during addition of paraquat, oxygen which is needed to generate ROS from paraquat. We demonstrated in this study that CydAB is effectively involved in O_2_ consumption (see further down). Moreover, the expression of *cydA* was not affected by paraquat, in the conditions that we used to monitor *sodB* expression (**Fig 4B**).

#### Peroxide stress

**Fig 5:**
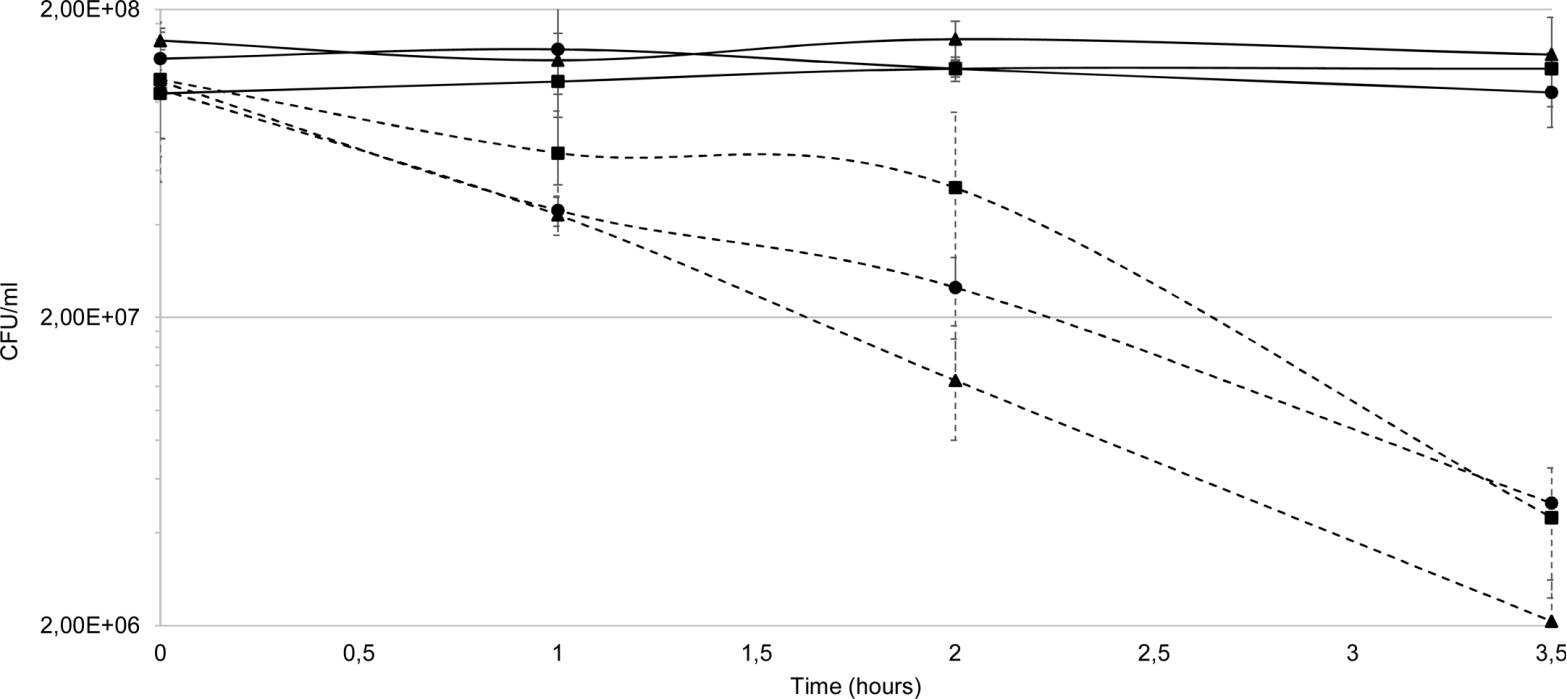
Survival assay with hydrogen peroxide. The effect of H_2_O_2_ on bacterial viability was assayed by counting the colony-forming units after exposing the cultures to 500 μM of H_2_O_2_ for 3.5 hours, in enriched BHI broth at 37°C in anaerobic conditions. Broken lines represent the numeration of viable cells after H_2_O_2_ addition (500 μM) and solid lines represent the control condition without H_2_O_2_. Wild-type (■), *cydAB* mutant (▲) and complemented *cydAB* mutant (●). Bars represent standard errors.

The absence of *cydAB* increased the susceptibility of exponentially growing *P*. *gingivalis* to 500 μM of H_2_O_2_ (Fig 5) while no difference was observed between wild-type and *cydAB* mutant when H_2_O_2_ was added to cultures in the stationary phase of growth (data not shown). This result is consistent with our previous data demonstrating that *cydAB* genes were expressed in the exponential phase of growth, displayed in Fig 2. However, the complementation of *cydAB* mutant with the native *cydAB* genes in *trans* only partially restored the resistance to H_2_O_2_ in the exponential phase: after 2 h exposure to 500 μM H_2_O_2_, the survival rate was 44.5% for wild-type, 21% for the complemented mutant, and 10% for the mutant (Fig 5). The susceptibility of *P*. *gingivalis* to H_2_O_2_ was significantly increased in the stationary phase compared to the exponential phase, with the survival rate dropping to 0.05% after two hours of treatment (data not shown).

### CydAB is involved in O_2_ consumption

**Fig 6:**
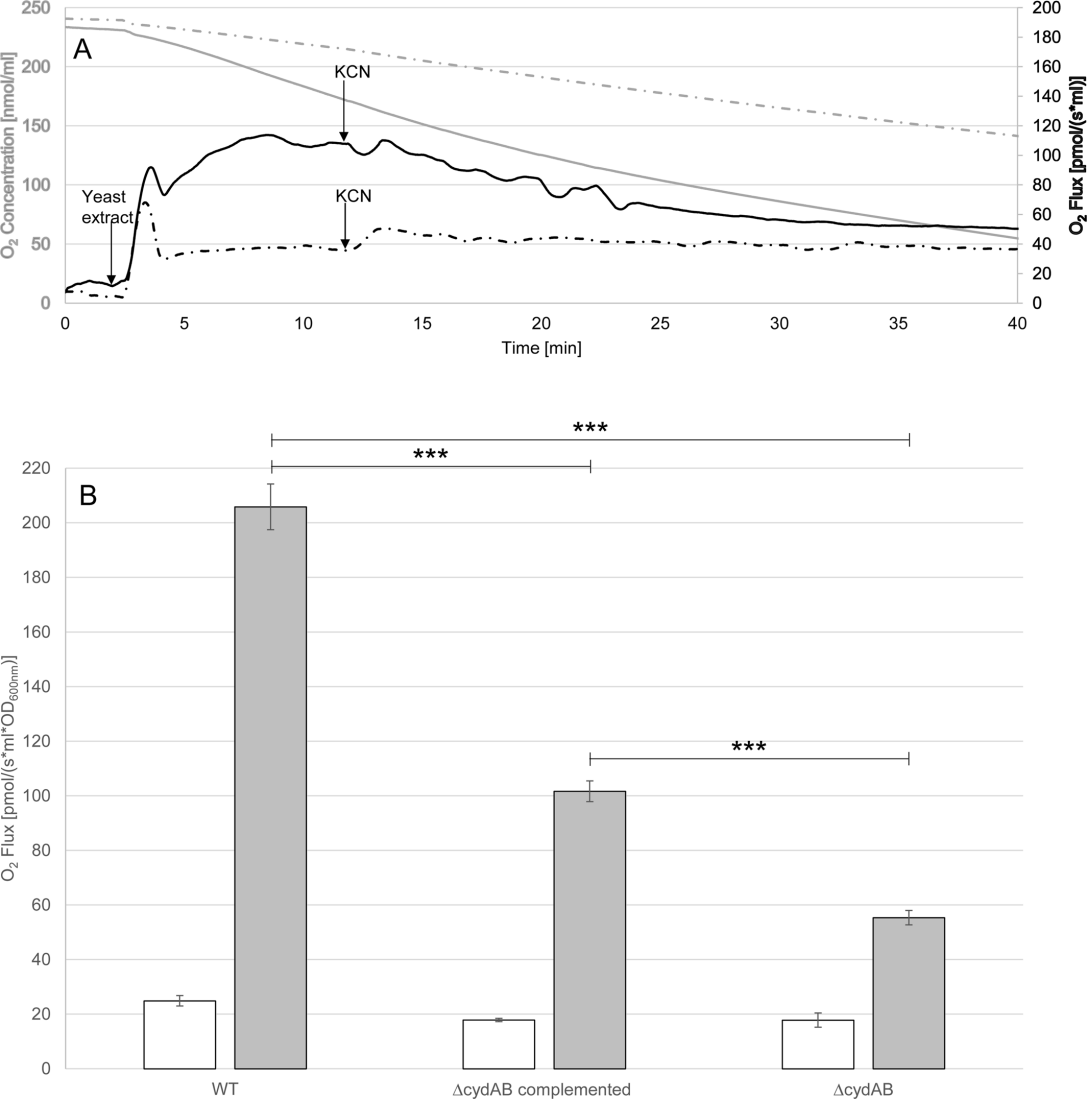
O_2_ consumption measured by high resolution respirometry. **(A)** The graphs represent the speed of consumption of O_2_ (O_2_ Flux per Volume) (black lines) and remaining exogenous O_2_ concentrations in the oxygraph chamber (grey lines) for both strains in PBS: wild-type (solid lines) and *cydAB* mutant (broken lines). OD_600 nm_ for wild-type and *cydAB* mutant were respectively of 0.588 and 0.544. Yeast extract (5 g/l) and potassium cyanide (KCN; 1 mM) addition were added at 2.5 min and 12 min respectively. **(B)** The effect of addition of yeast extract in PBS for O_2_ consumption is represented as the average and standard deviation of speed consumption of O_2_ (O_2_ Flux per Volume per OD_600 nm_) for each strain. The white histogram corresponds to the condition without yeast extract, and the grey histogram to the condition with yeast extract (5 g/l). Bars represent standard errors. Bars represent standard errors and asterisks indicate statistically significant results (*** p<0.0001). Statistical analysis were performed with GraphPad Prism software (California, USA). The Mann-Whitney test was used and p-values<0.05 were considered significant.

Despite no significant growth in microaerobic (6% O_2_ generated by campygen microaerophilic gas generator from Oxoid) and aerobic atmospheres in enriched BHI (data not shown), *P*. *gingivalis* ATCC 33277 can survive in oxygenated environments. We wanted to analyse the role of CydAB in O_2_ consumption in *P*. *gingivalis*. We were unable to detect any effect of the *cydAB* mutation on the instant respiration of enriched BHI-grown cells by high resolution respirometry (data not shown). However, O_2_ consumption at a rate of 18 pmol/(s*ml) was observed with non-inoculated enriched BHI medium (**S3A Fig**). To discard the effect of enriched BHI on O_2_ consumption, the cells were harvested by centrifugation and then washed twice in phosphate buffered saline (non O_2_-consuming **S3A Fig**). Neither the wild-type, nor the *cydAB* mutant or complemented mutant displayed a significant O_2_ consumption in PBS, with basal O_2_ consumption rates of respectively 17.8, 11.7, 11.4 pmol/(s*ml). This reduced O_2_ consumption is probably due to the lack of endogenous electron donors in washed cells (PBS). We found that respiration was reinstated in the wild-type by adding yeast extract to the PBS, while no effect was detected in the non-inoculated medium (**S3B Fig**). In this condition, the medium rate of O_2_ consumption was significantly lower in the *cydAB* mutant [35 pmol/(s*ml)] compared to WT [147 pmol/(s*ml)] and complemented mutant [67 pmol/(s*ml)] (**Fig 6A and 6B**). Interestingly, the addition of KCN (1 mM) decreased, but did not totally end, O_2_ consumption by wild-type but had no effect on the *cydAB* mutant (**Fig 6A**), confirming that the O_2_ consumption by the wild-type was mainly mediated by a cyanide-sensitive oxidase such as the cytochrome *bd* oxidase.

We have measured the intracellular concentrations of ATP in the media (enriched BHI or PBS plus yeast extract) that we used to monitor the O_2_ consumption, both in presence and absence of oxygen. No difference was observed with or without the *cydAB* genes, as depicted in **S4 Fig.**

### Cytochrome *bd* oxidase contributes to the dioxygen tolerance of *P*. *gingivalis*

**Fig 7:**
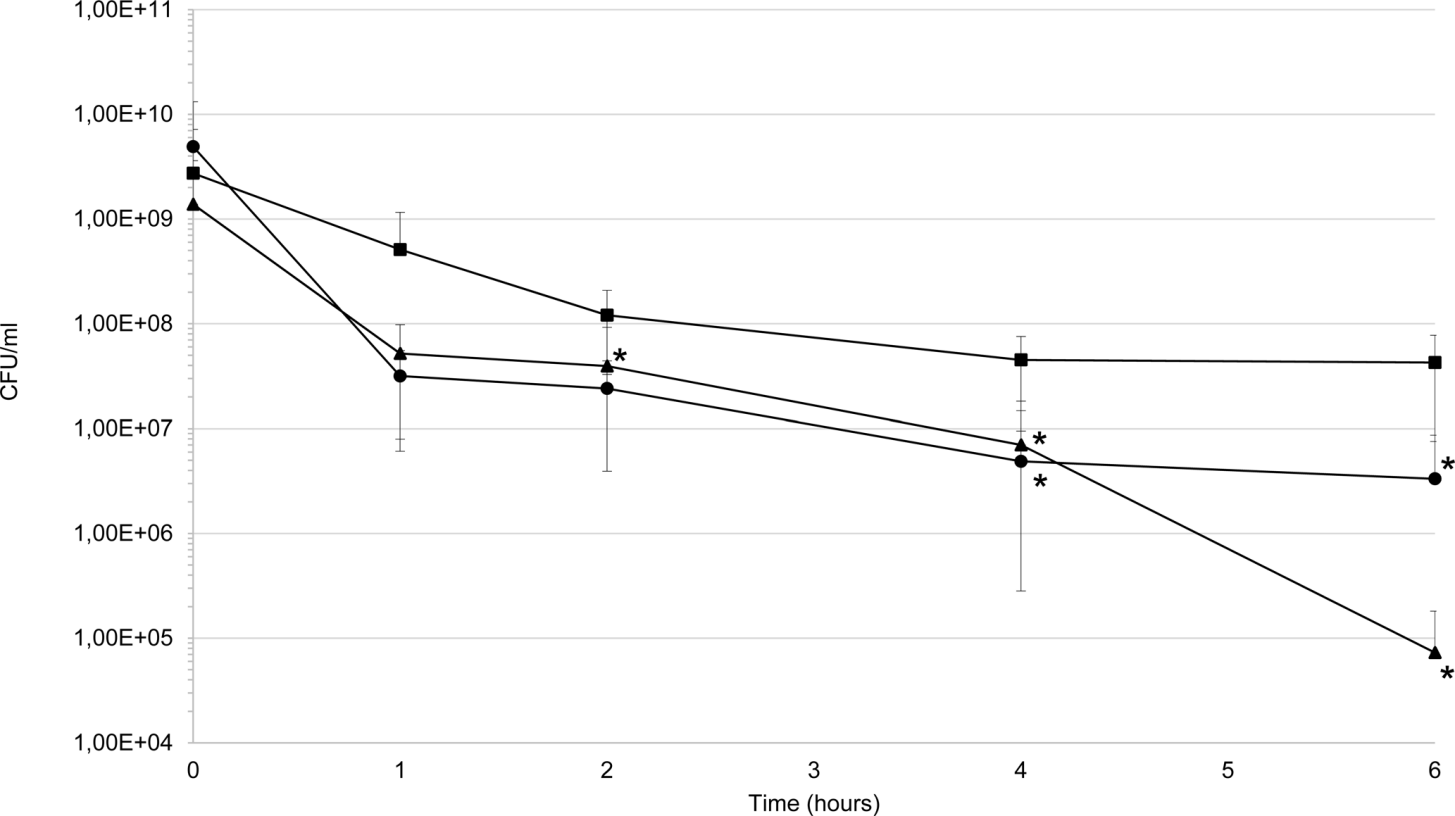
Dioxygen tolerance. The resistance to dioxygen was assayed by counting colony-forming units of bacterial cells suspended in PBS with yeast extract (5 g/l) and maintained in an aerobic atmosphere for six hours. Wild type (■), *cydAB* mutant (▲) and complemented *cydAB* mutant (●). Bars represent standard errors and asterisks indicate statistically significant differences between values of wild-type strain and mutant or complemented mutant strains (* p<0.05). Statistical analysis were performed with GraphPad Prism software (California, USA). The Mann-Whitney test was used and p-values<0.05 were considered significant.

To determine whether *cydAB* genes are required for dioxygen tolerance, wild-type, *cydAB* mutant and complemented *cydAB* mutant were exposed for six hours to atmospheric oxygen, in the PBS-yeast extract conditions for which CydAB dependent O_2_-consumption was detected. Unlike the control assays in anaerobic conditions, the *cydAB* mutant displayed a lower survival rate than the wild-type and complemented mutant in aerobic conditions, as depicted in **Fig 7**. However, 0.01% of *cydAB* mutant cells survived after six hours exposure to an aerobic atmosphere, indicating that CydAB is not the only factor contributing to the aerotolerance of *P*. *gingivalis* (**Fig 7**).

### Adhesion to and invasion of epithelial cells by *P*. *gingivalis* are not affected by *cydAB* mutation

**Fig 8:**
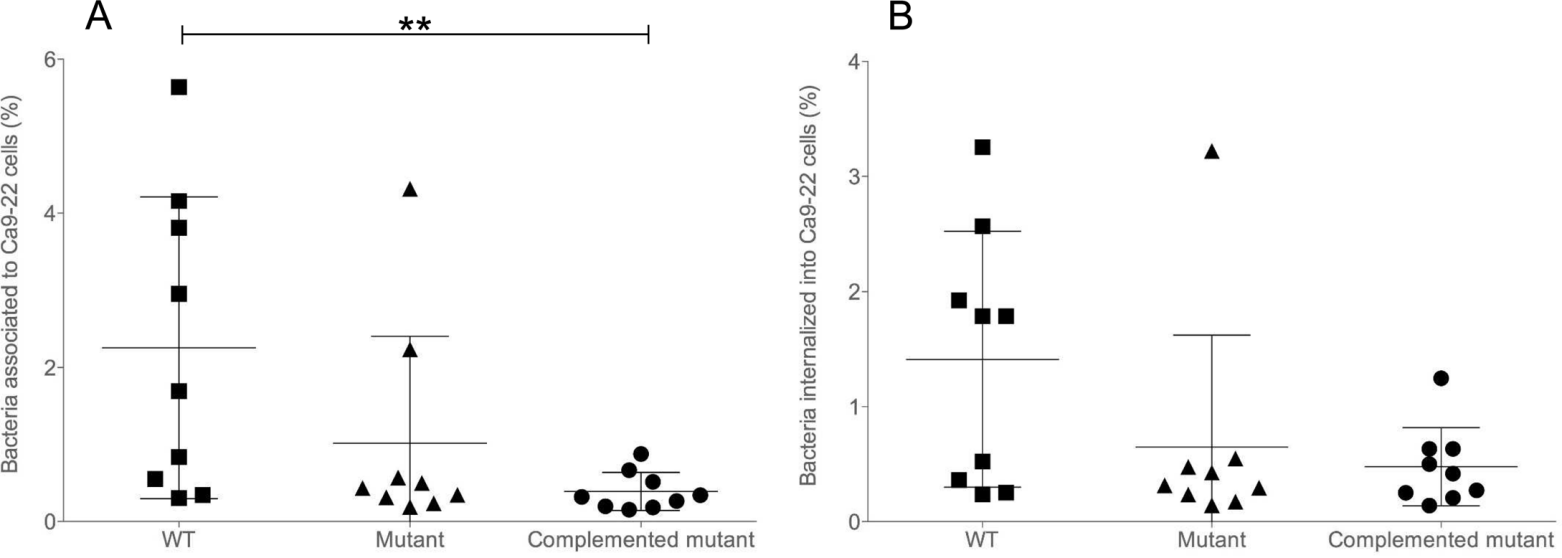
Adhesion and invasion to Ca9-22 epithelial cells. The percentage (vs. inoculum) of **(A)** adherent external and internal bacteria (“associated”), or **(B)** internal bacteria, which are remaining after incubation with Ca9-22 was assayed by counting colony forming units of bacterial cells per millilitre. Wild-type (■), *cydAB* mutant (▲) and complemented *cydAB* mutant (●). Bars represent mean with standard errors and asterisks indicate statistically significant differences between values of wild-type strain, mutant and complemented mutant strains (** p<0.01).

The *P*. *gingivalis cydAB* mutant and wild type displayed similar efficiency to adhere and/or invade the Ca9-22 gingival epithelial cells in aerobic atmosphere plus 5% CO_2_, as shown in **Fig 8A** which displayed all the Ca9-22-associated (inside and outside) bacteria. It is noteworthy that the efficiency of the complemented *cydAB* mutant, which overexpressed the *cydWAB* operon (**Fig 2**), was significantly altered (**Fig 8A**). When only the number of invasive bacterial cells was monitored, no difference was observed between the strains (**Fig 8B**). Survival assays with THP1 macrophages were also performed and neither the *cydAB* mutant nor the complemented mutant behaved differently than the WT (**S5 Fig**).

The presence of the cytochrome *bd* oxidase does not seem to be crucial for the interaction of *P*. *gingivalis* with host cells although the behavior of the overexpressing complemented strain requires further investigations.

## Conclusion

The *cydA* gene was identified in genomes of prokaryotes that have been classified as strict anaerobes, such as members of the *Archaeoglobus*, *Methanosarcina*, *Geobacter*, *Desulfovibrio* genera and members of the Bacteroides class [22]. *P*. *gingivalis*, a bacteroidetes, is considered as an obligatory anaerobic bacterium, although possessing an oxygen-dependent enzyme CydAB and an oxygen-generating superoxide dismutase SodB. The role of CydAB in O_2_ consumption and growth at nanomolar concentrations of oxygen was first demonstrated in the anaerobic bacterium *Bacteroides fragilis*, which was therefore classified as a nanaerobe [9]. This study is the first to evaluate the physiological role of CydAB in relation to oxygen species in the oral pathogen *P*. *gingivalis*.

This study shows that the cytochrome *bd* oxidase of *P*. *gingivalis* is necessary to confer an optimal resistance to hydrogen peroxide and superoxide. *P*. *gingivalis* is an obligate anaerobe but it is also known as an oxygen-tolerant organism. *P*. *gingivalis* possesses several systems that work against oxidative stress damage, including antioxidant enzymes [7], DNA binding proteins [33] and a heme layer that may act as an oxidative sink [34, 35], which altogether were suspected to account for its tolerance to dioxygen. We demonstrated in this study that CydAB of *P*. *gingivalis* was also involved in O_2_ consumption_,_ and in dioxygen resistance. Lewis *et al*. [20] and Diaz *et al*. [36] showed that *P*. *gingivalis* strains W83 and W50 were able to growth in a microaerobic atmosphere in the presence of excess hemin in medium (1 to 5 mg/l). However, we repeatedly failed to obtain visible growth of *P*. *gingivalis* ATCC 33277 in a microaerobic atmosphere (data not shown). Therefore, it is still unknown whether this *P*. *gingivalis* strain can benefit from low concentration of O_2_ for growth, and whether or not CydAB is essential for O_2_-mediated development. Moreover, in *Bacteroides fragilis*, a mutation inactivating one single gene, the *oxe* gene, allowed the growth up to 2% O_2_ [37] Additional experiments are needed to further investigate the growth of *P*. *gingivalis* ATCC 33277 in tiddly-controlled nanomolar concentrations of O_2_ and to search for possible *oxe*-like mutations in O_2_ growing *P*. *gingivalis* strains.

In conclusion, CydAB, which promotes ROS resistance, may confer a competitive advantage to *P*. *gingivalis*. Our data suggest that CydAB is a key element in the survival of *P*. *gingivalis* inside its ecological niche and to shape the oral community. Oral biofilms are constantly exposed to oxygen. Amazingly enough, most identified oral pathogens are classified as anaerobic bacteria. The survival of these oral bacterial species therefore depends on their specific tolerance to oxygen and the local oxygen tension inside the biofilm community. Cooperation also plays a role in shaping the community. For example, *Fusobacterium nucleatum* supports *P*. *gingivalis* growth by providing a capnophilic environment when growing in an oxygenated and CO_2_ depleted environment [38]. Competition between species is also relevant in oral biofilms: *Streptococcus sanguinis* or *Streptococcus gordonii* excrete H_2_O_2_ to compete with other species [39, 40]. *P*. *gingivalis* is often detected together with *Treponema denticola* in subgingival plaque. It is hypothesized that *T*. *denticola* can produce succinic acid, which is utilised by *P*. *gingivalis* in the cell envelope, while *P*. *gingivalis* produces isobutyric acid which increases *T*. *denticola* growth [41, 42]. Decreasing the local O_2_ concentration via high affinity cytochrome *bd* oxidase may favour the co-culture of associated anaerobic pathogens.

## Supporting Information

S1 Fig Co-occurrence of *cydWAB* genes. Taxa tree showing conserved operon architecture and co-occurrence of *cydWAB* genes. The analysis was performed with the String online software (Jensen *et al*. Nucleic Acids Res. 2009,37:D412-6) and with *P*. *gingivalis* ATCC 33277 *cydA* sequence as anchor. This neighborhood view shows runs of genes that occur repeatedly in close neighborhood in prokaryotic genomes, correlated with taxa tree. + indicated when sub-level species have been collapsed to reduce the taxa tree. For collapsed levels, the organism names are in green. Genes located together in a run are linked with a black line. The maximum allowed intergenic distance for genes to be considered as neighbor is 300 base pairs. Same color represents homologues based on amino-acid sequence similarity. Only predicted functional partners are displayed. PGN_1039 (light blue), PGN_1043 (purple) and PGN_1044 (dark blue) products appeared for *Porphyromonas* genus because the co-occurrence on the same DNA region of their coding genes in this genus is considered by the software as a parameter for a potential functional partnership.
(PDF)

S2 Fig RT-PCR on PGN_1039, *cydW*, *cydA*, *cydB* and PGN-1043. PCR was performed on cDNA obtained by reverse transcription of total RNA extracted from *P*. *gingivalis* ATCC 33277 (wild-type) with the following primers: (A) PGN_1039-L, TTCAGCCATACGCATCTGAG / PGN_1039-R, GTTGAATGCCACAATGTTCG for PGN_1039 gene; (B) PGN_1040-L, TCATGCGTATAGCTCGCTTTT / PGN_1040-R, TACCGACCTGTTGCTTCAGA for *cydW* gene; (C) PGN_1041-L, CCGGTAGGAATGACCTTCAA / PGN_1041-R, ATCCTTTCGCAGCAGGTAGA for *cydA* gene; (D) PGN_1042-L, TGGTAATGTATGGGGGAGGA / PGN_1042-R, GAGAACCACGTTCCACAGGT for *cydB* gene; (E) PGN_1043-L, GTCCCGACATCATAGCAGGT / PGN_1043-L, CAAGGTCCGTTGCCACTATT for PGN_1043 gene. RNA extract was used as negative control (-).The ladder (L) is the DNA Molecular Weight Marker VIII (Roche).
(PDF)

S3 Fig O2 consumption in BHI-enriched medium and in PBS. (**A**) O2 Flux per Volume is represented by black lines and O_2_ concentration is represented by grey lines for both media: PBS (solid lines) and enriched BHI (dashed lines). (**B**) The graphs represent the speed of consumption of O_2_ (O_2_ Flux per Volume) (black lines) by PBS and exogenous remaining O_2_ concentrations in the oxygraph chamber (grey lines). Yeast extract (5 g/l) was added at 9 min.
(PDF)

S4 Fig Intracellular ATP content. ATP content in *P*. *gingivalis* (wild-type, *cydAB* mutant and *cydAB* complemented mutant) was quantified by the BacTiter-Glo™ Microbial Cell Viability Assay kit (Promega). Histograms represent the concentration of ATP for 10^7^ bacteria and bars represent standard errors. *P*. *gingivalis* strains were grown overnight in enriched BHI at OD_600 nm_ of 0.5 (exponential phase). Samples were used to inoculate an enriched BHI medium to an OD_600 nm_ of 0.1 (for **A** and **B** experiments) or were washed and suspended in PBS supplemented with 5 g/l of yeast extract to an OD_600 nm_ of 0.1 (for **C** and **D** experiments). Each culture was split in two, one half was incubated for 7 hours in anaerobic conditions (for **A** and **C** experiments) and the other half was incubated for 5 hours in anaerobic conditions and shifted to micro-aerobic conditions for 2 hours (for **B** and **D** experiments).
(PDF)

S5 Fig Survival assay in presence of THP1 macrophages. Survival of *P*. *gingivalis* ATCC 33277 was assayed by counting colony forming units of bacterial cells per millilitre after 1 and 2 hours in presence of THP1 macrophages (JCRB cell bank, Japan). Wild-type (■), *cydAB* mutant (▲) and complemented *cydAB* mutant (●). Bars represent standard errors. THP1 monocyte were seeded into 24-well culture plates at a density of approximately 1,5.10^5^ cells per well and were differentiate to macrophages after 72 hours in aerobic conditions at 37°C with 5% CO_2_ in humidified atmosphere in Roswell Park Memorial Institute-1640 medium (RPMI-1640) enriched with 10% (v/v) fetal calf serum, 1% (v/v) of 200 mM L-glutamine, 1% (v/v) of 100 nM pyruvate sodium, 1% (v/v) of antibiotic mixture (10 U/μl penicillin and 10 U/μl streptomycin), 2% (v/v) of 1 M HEPES and 10 ng/ml of phorbol 12-myristate 13-acetate (PMA). Cells were washed with 500 μl of PBS and *P*. *gingivalis* strains (wild-type, *cydAB* mutant and *cydAB* complemented mutant) were added to THP1 macrophages with a multiplicity of infection of about 1:4000, in enriched RPMI-1640 without antibiotic mixture nor PMA. Plates were centrifuged for 5 minutes at 1000 g to promote bacteria-cell contact. Plates were incubated for 1 or 2 hours in aerobic conditions at 37°C with 5% CO_2_ in humidified atmosphere. Unattached bacteria were removed by three PBS washes. Samples were plated on Colombia agar supplemented with blood. Colonies were enumerated after 5 days of anaerobic incubation at 37°C. At least three independent experiments, each in duplicate, were conducted.
(PDF)

## Abbreviations

ROS: reactive oxygen species
RNS: reactive nitrogen species
