# Correction: The Cytochrome *bd* Oxidase of *Porphyromonas gingivalis* Contributes to Oxidative Stress Resistance and Dioxygen Tolerance

**DOI:** 10.1371/journal.pone.0145804

**Published:** 2015-12-21

**Authors:** The PLOS ONE Staff

The affiliations for the fifth and sixth authors are incorrect. Drs. Vincent Meuric and Martine Bonnaure-Mallet are not affiliated with # 2 but with #1 EA1254 Microbiologie—Risques Infectieux, University of Rennes1, Rennes, France, and #3 CHU Rennes, Rennes, France.

There are errors in the captions to Figs 2 and 4. Please view the complete, correct Fig 2 and Fig 4 here.

**Fig 2:**
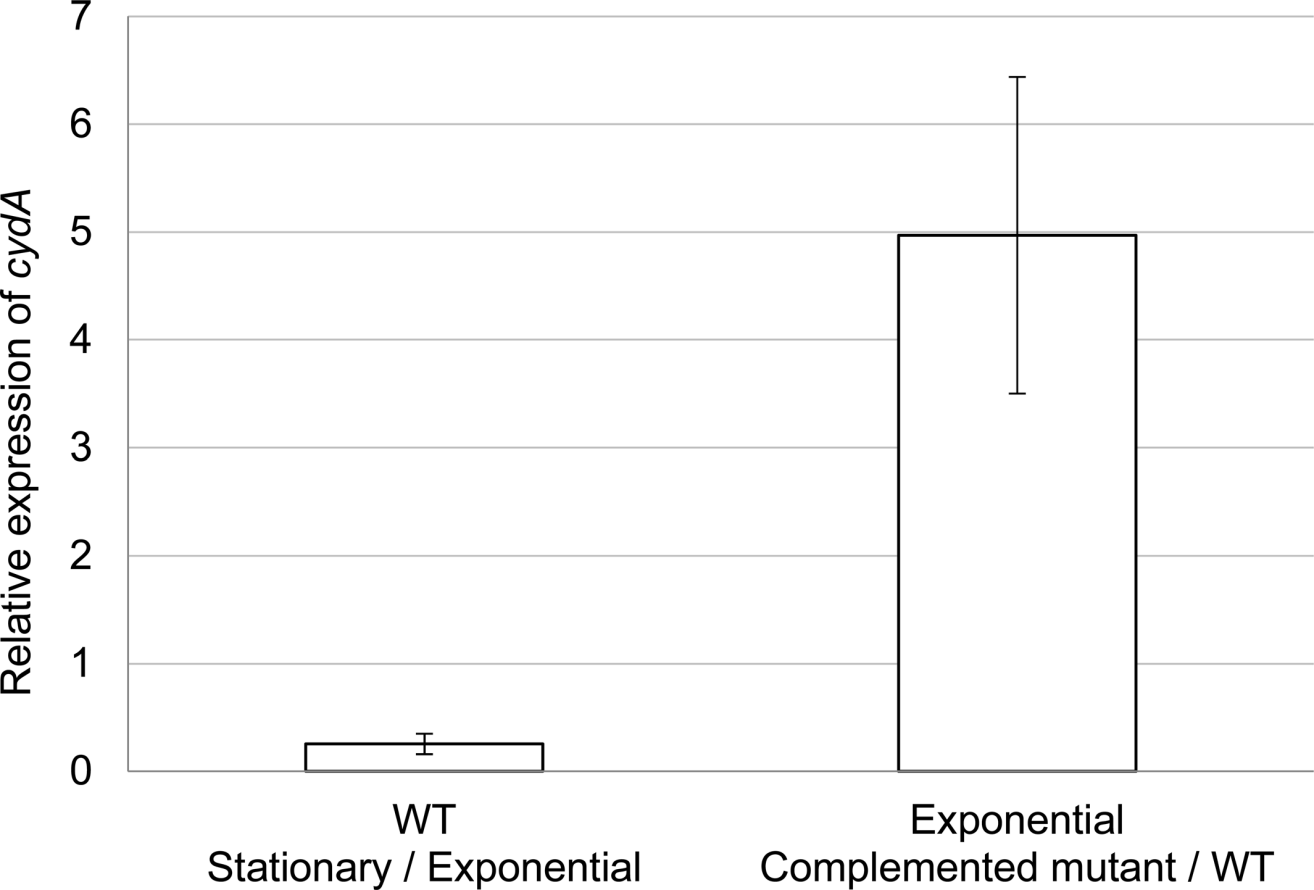
Relative expression of *cydA*. The relative expression was quantified by qRT-PCR with the 2^-ΔΔCt^ method. The normalization was done with the glucokinase house-keeping gene (PGN_0381). The expression of *cydA* in the wild-type strain was compared between stationary and exponential phases of growth. The white bar represents the ratio of expression in the stationary phase vs. the exponential phase. The relative expression of *cydA* in the exponential phase was compared between the complemented *cydAB* mutant and the wild type strains. The grey bar represents the ratio of expression of complemented mutant vs. wild-type. These data are the mean and standard deviations of two biological replicates containing three technical replicates.

**Fig 4:**
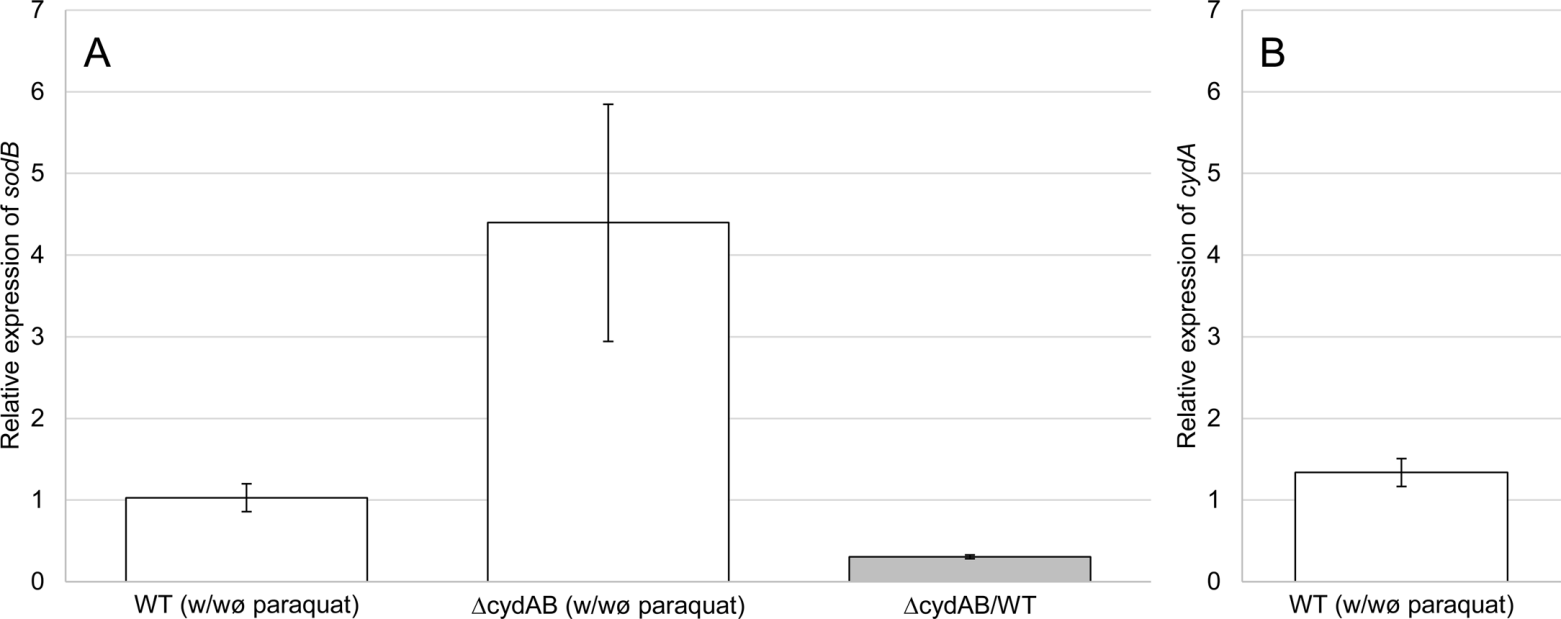
Relative expression of *sodB*. The relative expression of *sodB* was quantified by qRT-PCR with the 2^-ΔΔCt^ method. The normalization was done with the glucokinase gene (PGN_0381). The white histograms represent, for wild-type or *cydAB* mutant strains, the relative expression of *sodB* **(A)** or *cydA* **(B)** between both conditions: with paraquat (320 μM) and without paraquat (control condition). The grey histogram represents the relative expression of *sodB* between *cydAB* mutant and wild-type (control condition) in absence of paraquat. Bars represent standard errors.
