# Supplementary material for: The Cytochrome bd Oxidase of Porphyromonas gingivalis Contributes to Oxidative Stress Resistance and Dioxygen Tolerance
Source: PLoS One. 2015 Dec 2;10(12):e0143808. doi: 10.1371/journal.pone.0143808 (PMC4668044; doi:10.1371/journal.pone.0143808)
Supplement: S2 Fig — PCR was performed on cDNA obtained by reverse transcription of total RNA extracted from P. gingivalis ATCC 33277 (wild-type) with the following primers: (A) PGN_1039-L, TTCAGCCATACGCATCTGAG / PGN_1039-R, GTTGAATGCCACAATGTTCG for PGN_1039 gene; (B) PGN_1040-L, TCATGCGTATAGCTCGCTTTT / PGN_1040-R, TACCGACCTGTTGCTTCAGA for cydW gene; (C) PGN_1041-L, CCGGTAGGAATGACCTTCAA / PGN_1041-R, ATCCTTTCGCAGCAGGTAGA for cydA gene; (D) PGN_1042-L, TGGTAATGTATGGGGGAGGA / PGN_1042-R, GAGAACCACGTTCCACAGGT for cydB gene; (E) PGN_1043-L, GTCCCGACATCATAGCAGGT / PGN_1043-L, CAAGGTCCGTTGCCACTATT for PGN_1043 gene. RNA extract was used as negative control (-).The ladder (L) is the DNA Molecular Weight Marker VIII (Roche). (PDF) [file pone.0143808.s002.pdf]

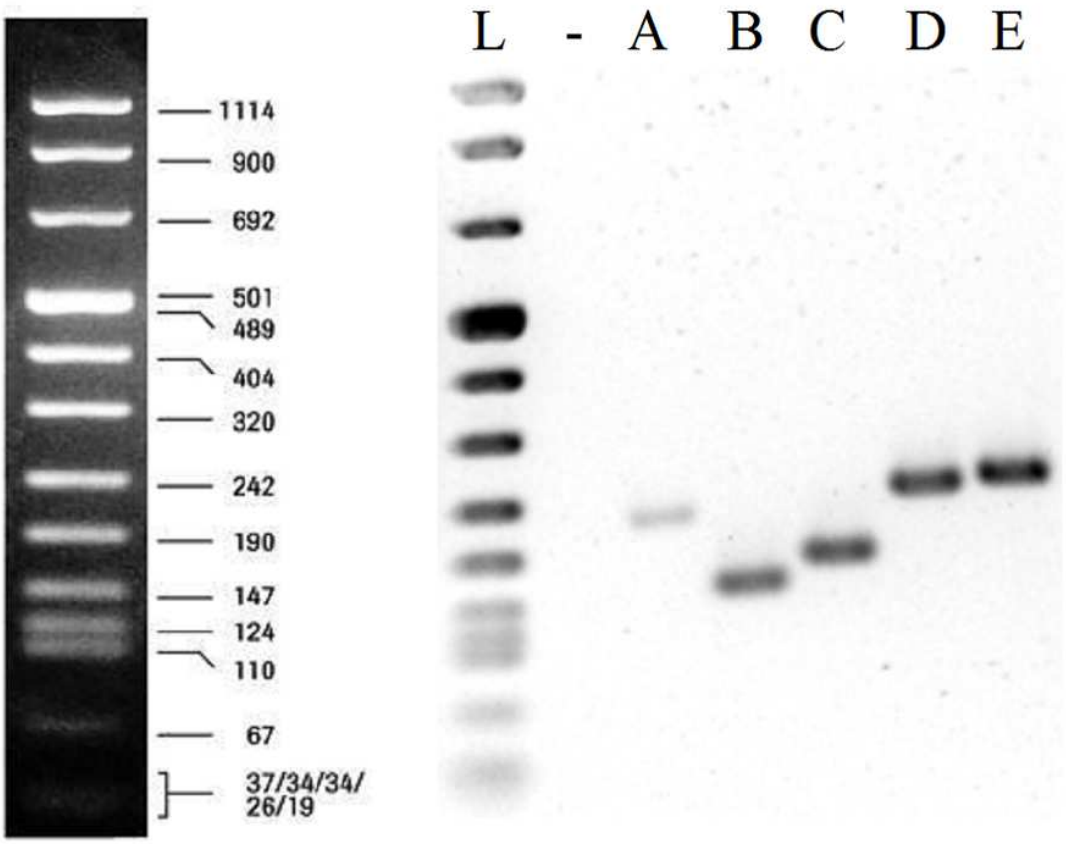

S2 Fig. RT-PCR on PGN_1039, *cydW, cydA, cydB* and PGN-1043.
PCR was performed on cDNA obtained by reverse transcription of total RNA extracted from *P. gingivalis* ATCC 33277 (wild-type) with the following primers: (**A**) PGN_1039-L, TTCAGCCATACGCATCTGAG / PGN_1039-R, GTTGAATGCCACAATGTTCG for PGN_1039 gene ; (**B**) PGN_1040-L, TCATGCGTATAGCTCGCTTTT / PGN_1040-R, TACCGACCTGTTGCTTCAGA for *cydW* gene ; (**C**) PGN_1041-L, CCGGTAGGAATGACCTTCAA / PGN_1041-R, ATCCTTTCGCAGCAGGTAGA for *cydA* gene ; (**D**) PGN_1042-L, TGGTAATGTATGGGGGAGGA / PGN_1042-R, GAGAACCACGTTCCACAGGT for *cydB* gene ; (**E**) PGN_1043-L, GTCCCGACATCATAGCAGGT / PGN_1043-L, CAAGGTCCGTTGCCACTATT for PGN_1043 gene. RNA extract was used as negative control (**-**).The ladder (**L**) is the DNA Molecular Weight Marker VIII (Roche).
