# Supplementary material for: The Cytochrome bd Oxidase of Porphyromonas gingivalis Contributes to Oxidative Stress Resistance and Dioxygen Tolerance
Source: PLoS One. 2015 Dec 2;10(12):e0143808. doi: 10.1371/journal.pone.0143808 (PMC4668044; doi:10.1371/journal.pone.0143808)
Supplement: S3 Fig — (A) O2 Flux per Volume is represented by black lines and O2 concentration is represented by grey lines for both media: PBS (solid lines) and enriched BHI (dashed lines). (B) The graphs represent the speed of consumption of O2 (O2 Flux per Volume) (black lines) by PBS and exogenous remaining O2 concentrations in the oxygraph chamber (grey lines). Yeast extract (5 g/l) was added at 9 min. (PDF) [file pone.0143808.s003.pdf]

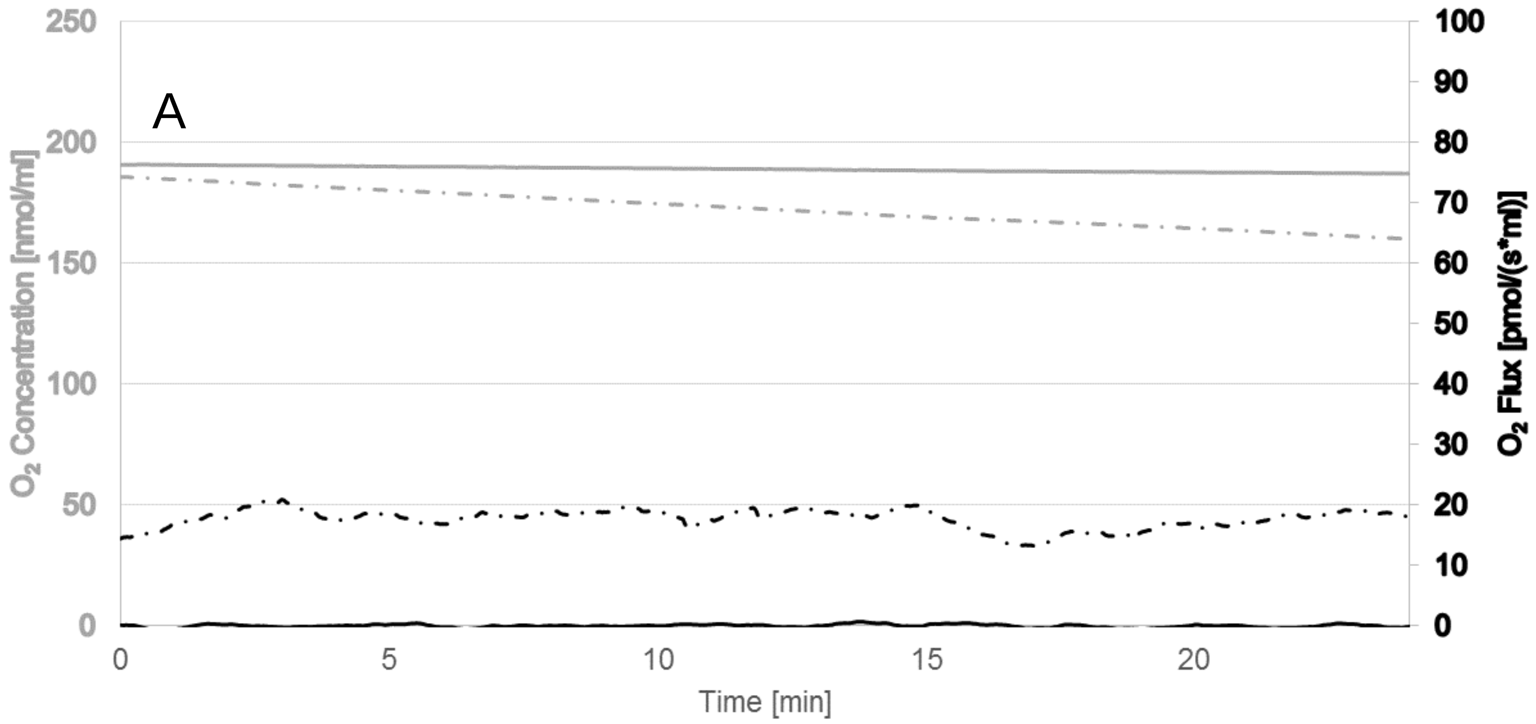

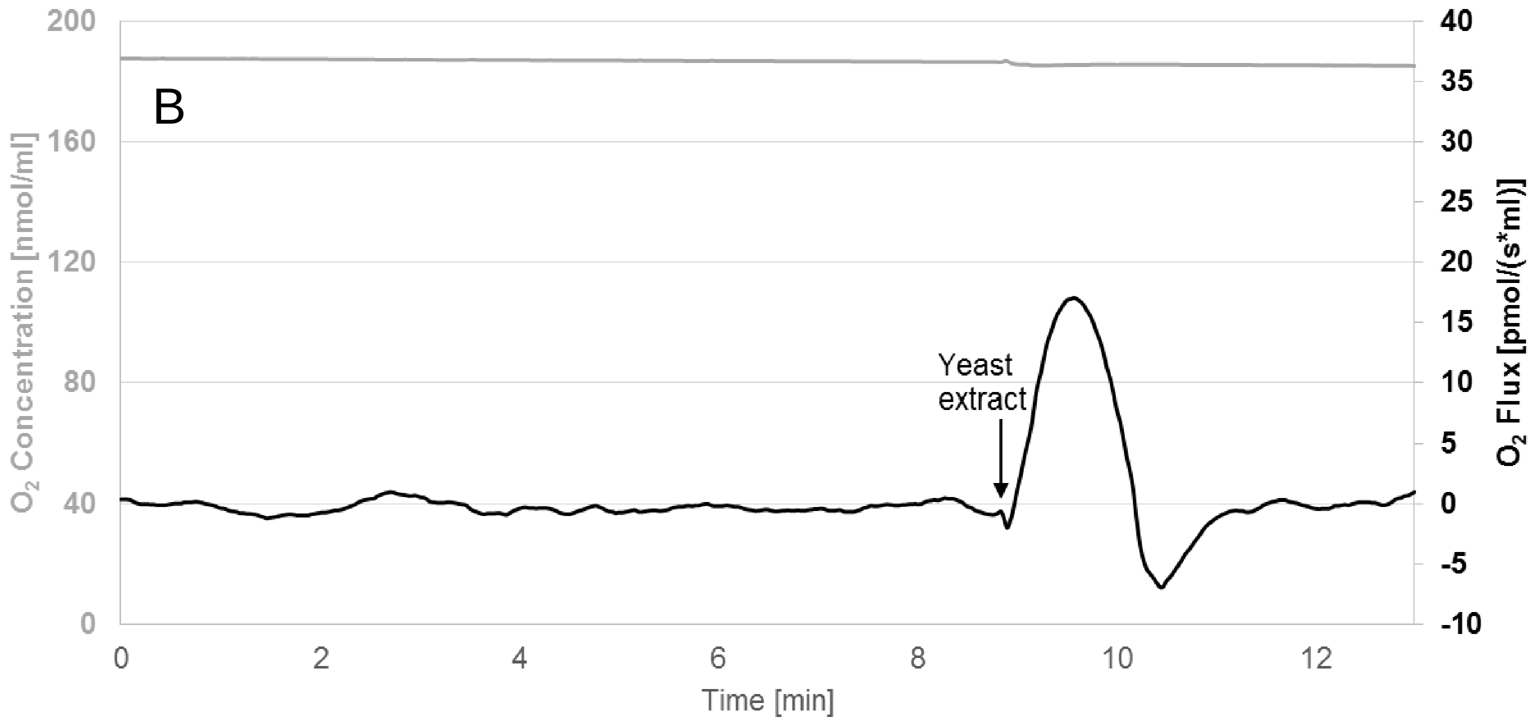

S3 Fig. O_2 consumption in BHI-enriched medium and in PBS.

(A) O_2 Flux per Volume is represented by black lines and O_2 concentration is represented by grey lines for both media : PBS (solid lines) and enriched BHI (dashed lines).

(B) The graphs represent the speed of consumption of O_2 (O_2 Flux per Volume) (black lines) by PBS and exogenous remaining O_2 concentrations in the oxygraph chamber (grey lines). Yeast extract (5 g/l) was added at 9 min.
