# Supplementary material for: The Cytochrome bd Oxidase of Porphyromonas gingivalis Contributes to Oxidative Stress Resistance and Dioxygen Tolerance
Source: PLoS One. 2015 Dec 2;10(12):e0143808. doi: 10.1371/journal.pone.0143808 (PMC4668044; doi:10.1371/journal.pone.0143808)
Supplement: S1 Fig — Taxa tree showing conserved operon architecture and co-occurrence of cydWAB genes. The analysis was performed with the String online software (Jensen et al. Nucleic Acids Res. 2009,37:D412-6) and with P. gingivalis ATCC 33277 cydA sequence as anchor. This neighborhood view shows runs of genes that occur repeatedly in close neighborhood in prokaryotic genomes, correlated with taxa tree. + indicated when sub-level species have been collapsed to reduce the taxa tree. For collapsed levels, the organism names are in green. Genes located together in a run are linked with a black line. The maximum allowed intergenic distance for genes to be considered as neighbor is 300 base pairs. Same color represents homologues based on amino-acid sequence similarity. Only predicted functional partners are displayed. PGN_1039 (light blue), PGN_1043 (purple) and PGN_1044 (dark blue) products appeared for Porphyromonas genus because the co-occurrence on the same DNA region of their coding genes in this genus is considered by the software as a parameter for a potential functional partnership. (PDF) [file pone.0143808.s001.pdf]

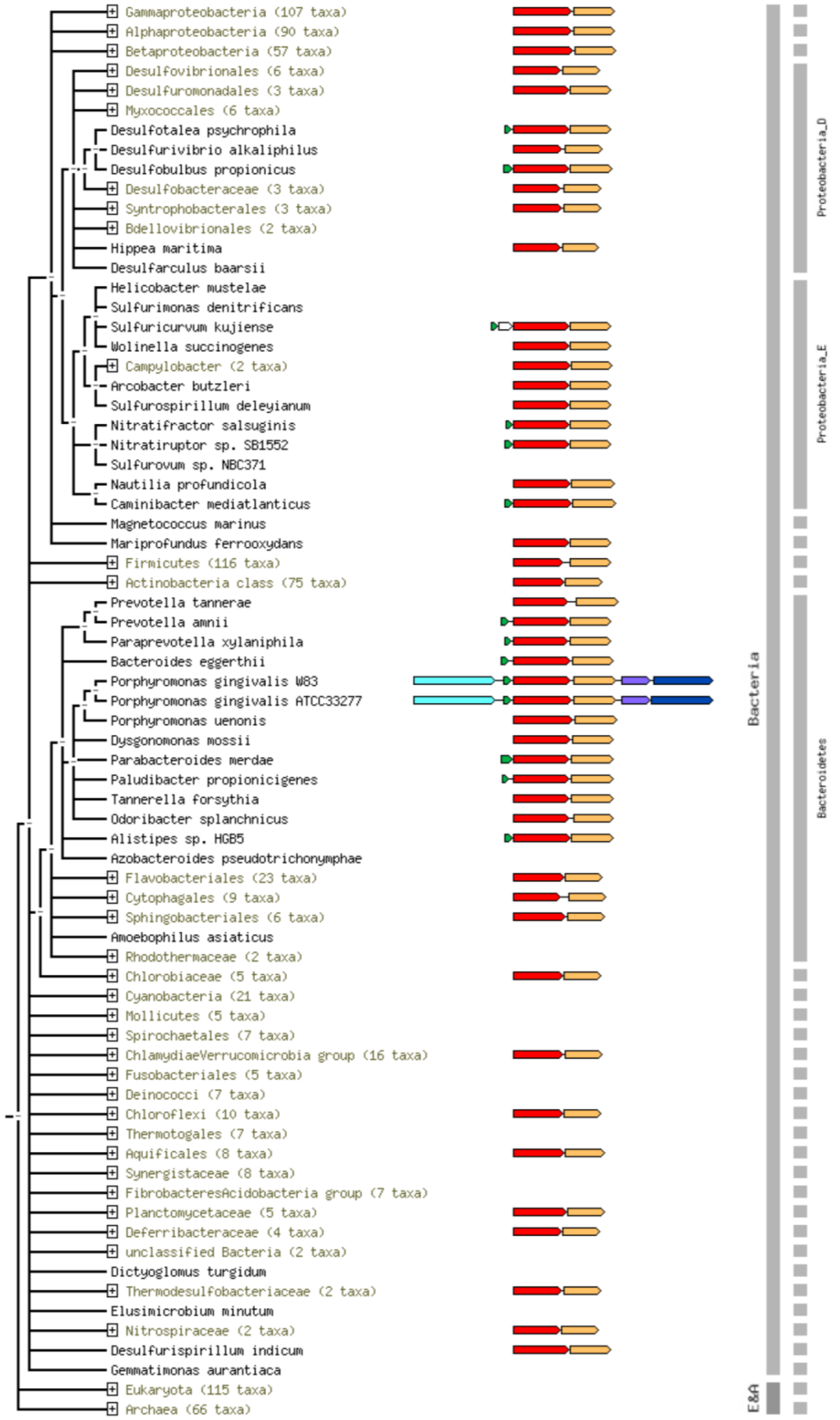


**S1 Fig. Co-occurrence of *cydWAB* genes.**

Taxa tree showing conserved operon architecture and co-occurrence of *cydWAB* genes. The analysis was performed with the String online software (Jensen *et al.* Nucleic Acids Res. 2009,37:D412-6) and with *P. gingivalis* ATCC 33277 *cydA* sequence as anchor. This neighborhood view shows runs of genes that occur repeatedly in close neighborhood in prokaryotic genomes, correlated with taxa tree. + indicated when sub-level species have been collapsed to reduce the taxa tree. For collapsed levels, the organism names are in green. Genes located together in a run are linked with a black line. The maximum allowed intergenic distance for genes to be considered as neighbor is 300 base pairs. Same color represents homologues based on amino-acid sequence similarity. Only predicted functional partners are displayed. PGN-1039 (light blue), PGN-1043 (purple) and PGN_1044 (dark blue) products appeared for *Porphyromonas* genus because the co-occurrence on the same DNA region of their coding genes in this genus is considered by the software as a parameter for a potential functional partnership.
