# Supplementary material for: The Cytochrome bd Oxidase of Porphyromonas gingivalis Contributes to Oxidative Stress Resistance and Dioxygen Tolerance
Source: PLoS One. 2015 Dec 2;10(12):e0143808. doi: 10.1371/journal.pone.0143808 (PMC4668044; doi:10.1371/journal.pone.0143808)
Supplement: S5 Fig — Survival of P. gingivalis ATCC 33277 was assayed by counting colony forming units of bacterial cells per millilitre after 1 and 2 hours in presence of THP1 macrophages (JCRB cell bank, Japan). Wild-type (■), cydAB mutant (▲) and complemented cydAB mutant (●). Bars represent standard errors. THP1 monocyte were seeded into 24-well culture plates at a density of approximately 1,5.105 cells per well and were differentiate to macrophages after 72 hours in aerobic conditions at 37°C with 5% CO2 in humidified atmosphere in Roswell Park Memorial Institute-1640 medium (RPMI-1640) enriched with 10% (v/v) fetal calf serum, 1% (v/v) of 200 mM L-glutamine, 1% (v/v) of 100 nM pyruvate sodium, 1% (v/v) of antibiotic mixture (10 U/μl penicillin and 10 U/μl streptomycin), 2% (v/v) of 1 M HEPES and 10 ng/ml of phorbol 12-myristate 13-acetate (PMA). Cells were washed with 500 μl of PBS and P. gingivalis strains (wild-type, cydAB mutant and cydAB complemented mutant) were added to THP1 macrophages with a multiplicity of infection of about 1:4000, in enriched RPMI-1640 without antibiotic mixture nor PMA. Plates were centrifuged for 5 minutes at 1000 g to promote bacteria-cell contact. Plates were incubated for 1 or 2 hours in aerobic conditions at 37°C with 5% CO2 in humidified atmosphere. Unattached bacteria were removed by three PBS washes. Samples were plated on Colombia agar supplemented with blood. Colonies were enumerated after 5 days of anaerobic incubation at 37°C. At least three independent experiments, each in duplicate, were conducted. (PDF) [file pone.0143808.s005.pdf]

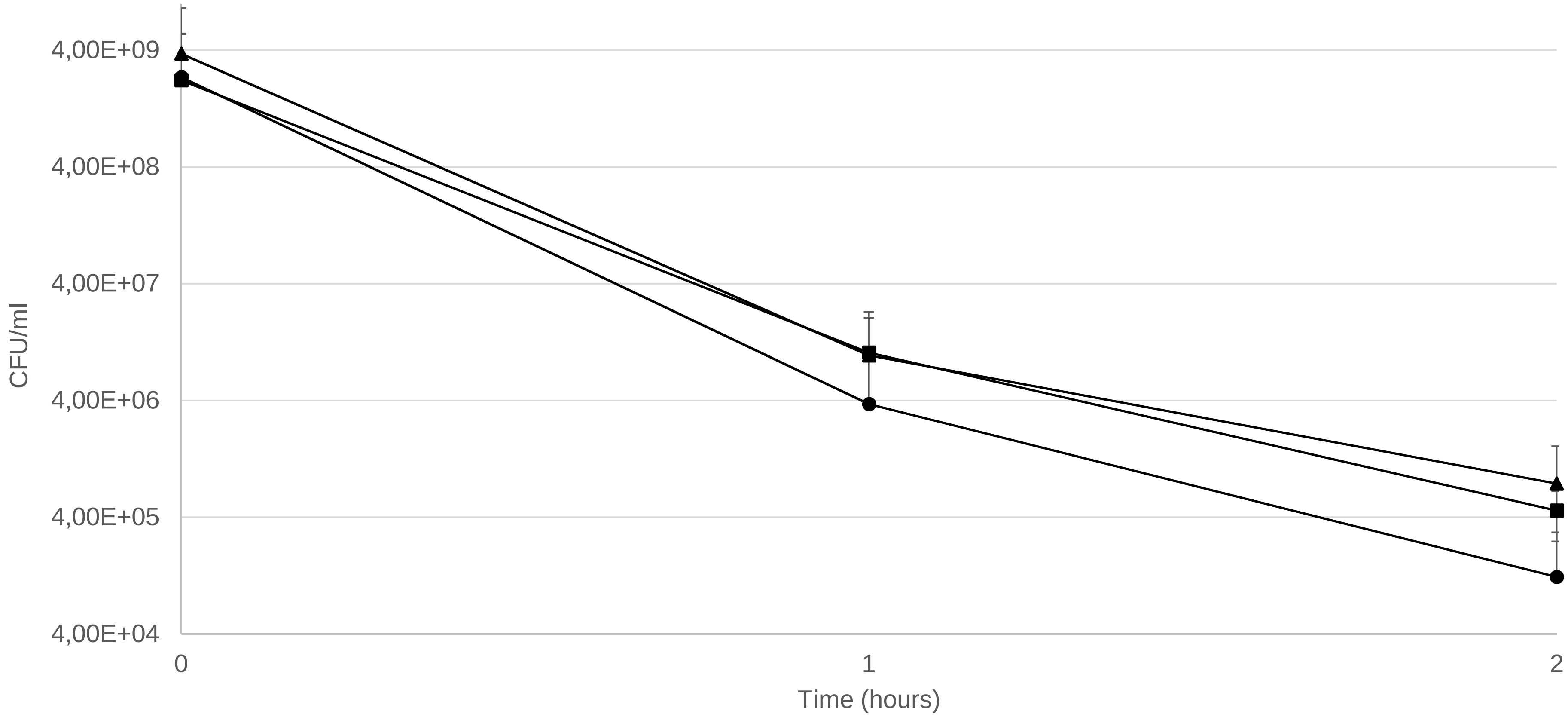

S5 Fig. Survival assay in presence of THP1 macrophages.

Survival of *P. gingivalis* ATCC 33277 was assayed by counting colony forming units of bacterial cells per millilitre after 1 and 2 hours in presence of THP1 macrophages (JCRB cell bank, Japan). Wild-type (■), *cydAB* mutant (▲) and complemented *cydAB* mutant (●). Bars represent standard errors. THP1 monocyte were seeded into 24-well culture plates at a density of approximately $1{,}5.10^5$ cells per well and were differentiate to macrophages after 72 hours in aerobic conditions at 37°C with 5 % CO2 in humidified atmosphere in Roswell Park Memorial Institute-1640 medium (RPMI-1640) enriched with 10 % (v/v) fetal calf serum, 1 % (v/v) of 200 mM L-glutamine, 1 % (v/v) of 100 nM pyruvate sodium, 1 % (v/v) of antibiotic mixture (10 U/µl penicillin and 10 U/µl streptomycin), 2 % (v/v) of 1 M HEPES and 10 ng/ml of phorbol 12-myristate 13-acetate (PMA). Cells were washed with 500 µl of PBS and *P. gingivalis* strains (wild-type, *cydAB* mutant and *cydAB* complemented mutant) were added to THP1 macrophages with a multiplicity of infection of about 1:4000, in enriched RPMI-1640 without antibiotic mixture nor PMA. Plates were centrifuged for 5 minutes at 1000 g to promote bacteria-cell contact. Plates were incubated for 1 or 2 hours in aerobic conditions at 37°C with 5 % CO_2 in humidified atmosphere. Unattached bacteria were removed by three PBS washes. Samples were plated on Colombia agar supplemented with blood. Colonies were enumerated after 5 days of anaerobic incubation at 37°C. At least three independent experiments, each in duplicate, were conducted.
