# Supplementary material for: The Cytochrome bd Oxidase of Porphyromonas gingivalis Contributes to Oxidative Stress Resistance and Dioxygen Tolerance
Source: PLoS One. 2015 Dec 2;10(12):e0143808. doi: 10.1371/journal.pone.0143808 (PMC4668044; doi:10.1371/journal.pone.0143808)
Supplement: S4 Fig — ATP content in P. gingivalis (wild-type, cydAB mutant and cydAB complemented mutant) was quantified by the BacTiter-Glo™ Microbial Cell Viability Assay kit (Promega). Histograms represent the concentration of ATP for 107 bacteria and bars represent standard errors. P. gingivalis strains were grown overnight in enriched BHI at OD600 nm of 0.5 (exponential phase). Samples were used to inoculate an enriched BHI medium to an OD600 nm of 0.1 (for A and B experiments) or were washed and suspended in PBS supplemented with 5 g/l of yeast extract to an OD600 nm of 0.1 (for C and D experiments). Each culture was split in two, one half was incubated for 7 hours in anaerobic conditions (for A and C experiments) and the other half was incubated for 5 hours in anaerobic conditions and shifted to micro-aerobic conditions for 2 hours (for B and D experiments). (PDF) [file pone.0143808.s004.pdf]

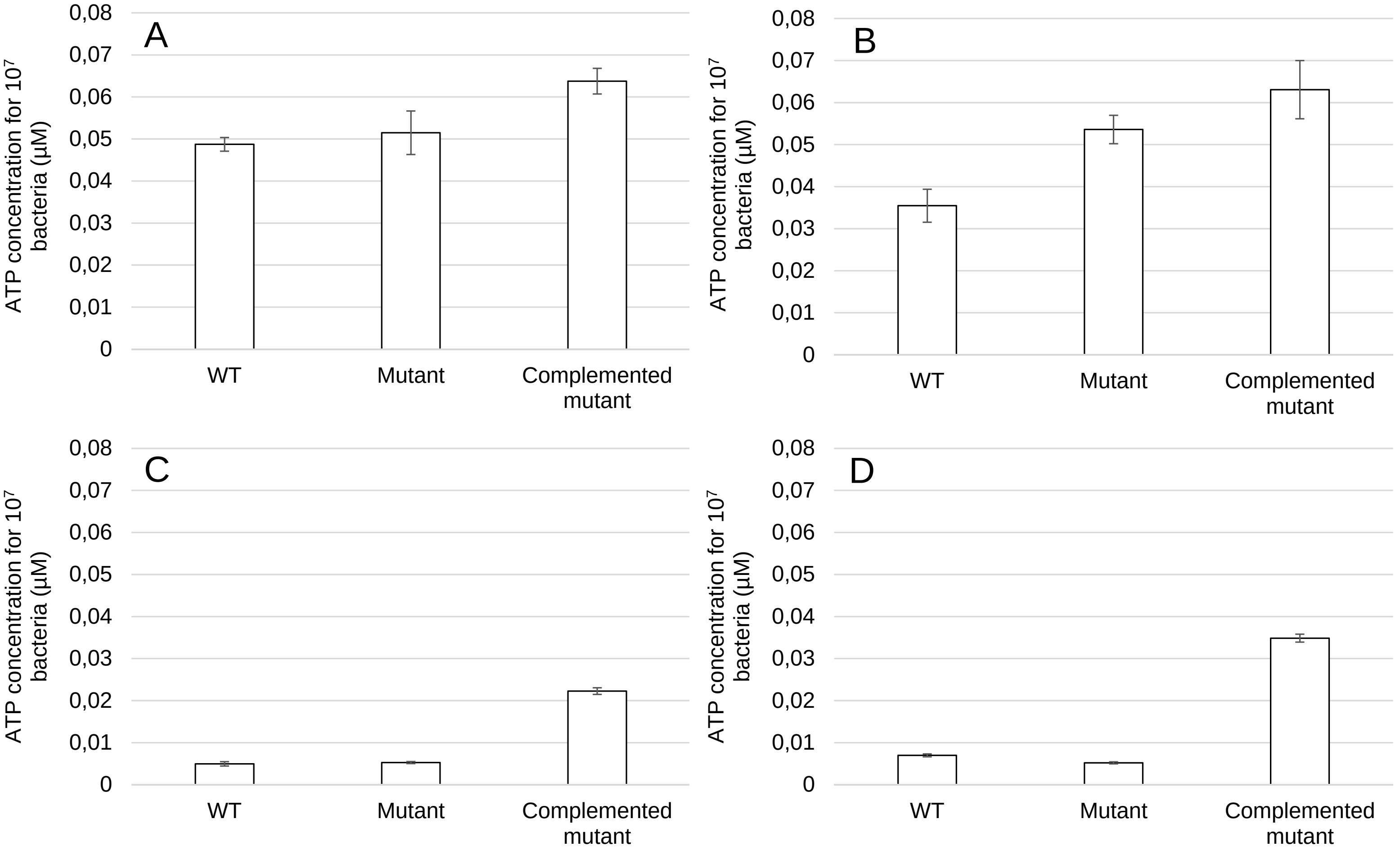

S4 Fig. Intracellular ATP content.

ATP content in *P. gingivalis* (wild-type, *cydAB* mutant and *cydAB* complemented mutant) was quantified by the BacTiter-Glo™ Microbial Cell Viability Assay kit (Promega). Histograms represent the concentration of ATP for 10^7 bacteria and bars represent standard errors. *P. gingivalis* strains were grown overnight in enriched BHI at $OD_{600\ nm}$ of 0.5 (exponential phase). Samples were used to inoculate an enriched BHI medium to an $OD_{600\ nm}$ of 0.1 (for **A** and **B** experiments) or were washed and suspended in PBS supplemented with 5 g/l of yeast extract to an $OD_{600\ nm}$ of 0.1 (for **C** and **D** experiments). Each culture was split in two, one half was incubated for 7 hours in anaerobic conditions (for **A** and **C** experiments) and the other half was incubated for 5 hours in anaerobic conditions and shifted to micro-aerobic conditions for 2 hours (for **B** and **D** experiments).
